# Development and Validation of a Nomogram for the Prediction of Hospital Mortality of Patients With Encephalopathy Caused by Microbial Infection: A Retrospective Cohort Study

**DOI:** 10.3389/fmicb.2021.737066

**Published:** 2021-08-19

**Authors:** Lina Zhao, Yun Li, Yunying Wang, Qian Gao, Zengzheng Ge, Xibo Sun, Yi Li

**Affiliations:** ^1^Emergency Department, State Key Laboratory of Complex Severe and Rare Diseases, Peking Union Medical College Hospital, Peking Union Medical College, Chinese Academy of Medical Sciences, Beijing, China; ^2^Department of Critical Care Medicine, Chifeng Municipal Hospital, Chifeng Clinical Medical College of Inner Mongolia Medical University, Chifeng, China; ^3^Department of Anesthesiology, Chifeng Municipal Hospital, Chifeng Clinical Medical College of Inner Mongolia Medical University, Chifeng, China; ^4^Department of Neurology, Yidu Central Hospital Affiliated to Weifang Medical University, Weifang, China

**Keywords:** sepsis associated encephalopathy, prognosis, hospital mortality, nomogram, microbial infection

## Abstract

**Background:**

Hospital mortality is high for patients with encephalopathy caused by microbial infection. Microbial infections often induce sepsis. The damage to the central nervous system (CNS) is defined as sepsis-associated encephalopathy (SAE). However, the relationship between pathogenic microorganisms and the prognosis of SAE patients is still unclear, especially gut microbiota, and there is no clinical tool to predict hospital mortality for SAE patients. The study aimed to explore the relationship between pathogenic microorganisms and the hospital mortality of SAE patients and develop a nomogram for the prediction of hospital mortality in SAE patients.

**Methods:**

The study is a retrospective cohort study. The lasso regression model was used for data dimension reduction and feature selection. Model of hospital mortality of SAE patients was developed by multivariable Cox regression analysis. Calibration and discrimination were used to assess the performance of the nomogram. Decision curve analysis (DCA) to evaluate the clinical utility of the model.

**Results:**

Unfortunately, the results of our study did not find intestinal infection and microorganisms of the gastrointestinal (such as: Escherichia coli) that are related to the prognosis of SAE. Lasso regression and multivariate Cox regression indicated that factors including respiratory failure, lactate, international normalized ratio (INR), albumin, SpO_2_, temperature, and renal replacement therapy were significantly correlated with hospital mortality. The AUC of 0.812 under the nomogram was more than that of the Simplified Acute Physiology Score (0.745), indicating excellent discrimination. DCA demonstrated that using the nomogram or including the prognostic signature score status was better than without the nomogram or using the SAPS II at predicting hospital mortality.

**Conclusion:**

The prognosis of SAE patients has nothing to do with intestinal and microbial infections. We developed a nomogram that predicts hospital mortality in patients with SAE according to clinical data. The nomogram exhibited excellent discrimination and calibration capacity, favoring its clinical utility.

## Introduction

Sepsis is defined as a life-threatening organ dysfunction with host response imbalance caused by infection (Singer et al., 2016). Sepsis-associated encephalopathy (SAE) is defined as diffuse brain dysfunction without the central nervous system (CNS) infection in sepsis patients. Metabolic encephalopathy, drug intoxication, structural brain lesions, cerebrovascular events, encephalitis, meningitis, and non-convulsive status epilepticus need to be ruled out in sepsis patients before a diagnosis of SAE (Eidelman et al., 1996). SAE develops in up to 70% of septic patients (Gofton and Young, 2012; Fraser et al., 2014).

SAE is related to increased mortality, extensive costs, prolonged hospitalization, followed by persistent cognitive impairment (Iwashyna et al., 2010; Sonneville et al., 2017). The mortality rates of SAE patients over 60% in sepsis patients (Eidelman et al., 1996; Schuler et al., 2018). At hospital discharge, 45% of patients are related to the development of dementia (Annane and Sharshar, 2015). Early recognition of brain injury and prompt management are of great importance for the survival and prognosis of septic patients. Intestinal microbial infection is one of the important sites of infection in patients with sepsis. Intestinal microbes are not only related to infections. Studies have found that can have an impact on the brain through the microbiota-gut-brain axis, included depression, anxiety, dementia, and other diseases (Grochowska et al., 2018). Li et al. (2018) found that intestinal flora can affect SAE through the vagus nerve. The relationship between intestinal flora and the prognosis of SAE patients is still unclear.

Therefore, further studies for identifying the relationship between intestinal flora and the prognosis of SAE patients, and the predictors of the prognosis of SAE patients, especially accurate and measurable prediction models for hospital mortality, are pivotal for risk-optimized therapeutic strategies and to improve the prognosis of sepsis patients. This study aimed to investigate the predictors associated with hospital mortality in patients with SAE and establish a comprehensive visual predictive nomogram of hospital mortality, calculating a probabilistic estimate that could be of use to clinicians these patients.

## Materials and Methods

### Data Source

Data were obtained from the Medical Information Mart for Intensive Care (MIMIC-III, Version 1.4), which contains 46,520 patients admitted to the Beth Israel Deaconess Medical Center (Boston, MA, United States) from 2001 to 2012 (Johnson et al., 2016). The database documents included charted events such as demographics, vital signs, microbiology events, medication prescriptions, laboratory tests, etc. International Classification of Diseases, Ninth Revision (ICD-9) codes were also documented by hospital staff on patient discharge. The following CITI program course was completed: CITI 33690380. The raw data were extracted using a structure query language (SQL) using Navicat and further processed with R software.

### Patient Population

Inclusion criteria were as follows: Patients with (1) sepsis 3.0. (2) age ≥ 18 years-old. (3) at least 24 h stay in the ICU. Sepsis was defined as an infected patient on discharge according to ICD-9 codes and microbial culture positive. According to the definition of sepsis 3.0, we included patients with SOFA score ≥ 2.

Exclusion criteria (Sonneville et al., 2017; Yang Y. et al., 2020): (1) Patients without SAE. (2) Primary brain injury including traumatic brain injury, intracerebral hemorrhage, cerebral embolism, ischemic stroke, epilepsy, or intracranial infection, and other cerebrovascular diseases according to ICD-9 codes. (Supplementary Materials 1–5); (3) Mental disorders and neurological disease (Supplementary Material 6); (4) Chronic alcohol or drug abuse (Supplementary Material 7); (5) Encephalopathy caused by other causes including metabolic encephalopathy, hepatic encephalopathy, hypertensive encephalopathy, hypoglycemic coma, and other liver disease or kidney disease affecting consciousness (Supplementary Material 8); (6) Severe electrolyte imbalances or blood glucose disturbances, including hyponatremia (<120 mmol/l), hyperglycemia (>180 mg/dl), or hypoglycemia (<54 mg/dl); (7) Partial pressure of CO_2_ (PCO_2_) ≥ 80 mmHg; (8)Without an evaluation of a Glasgow Coma Scale (GCS) score; (9) Patients who have been sedated by tracheal intubation at the time of admission.

### Sepsis-Associated Encephalopathy

Sepsis-associated encephalopathy was defined as (1) patients with GCS < 15. (2) The patient was diagnosed with delirium, cognitive impairment, altered mental status according to the ICD-9 code. (3) The patient was treated with haloperidol during hospitalization. (4) Exclude consciousness disorders caused by other reasons. Many studies use GCS score as an essential tool for evaluating SAE patients (Iwashyna et al., 2010).

### Data Extraction and Management

The following data was extracted from the MIMIC III database using R statistical software (R Foundation for Statistical Computing, Vienna, Austria): basic patient demographical data, vital signs (mean value) during the first 24 h of intensive care unit (ICU) stay, the first laboratory data since ICU admission and severity scores (including SAPS II, quick sequential organ failure assessment (qSOFA) score, sequential organ failure assessment (SOFA) score), comorbidity index at discharge according to the ICD-9 code (Supplementary Material 9), site of infection and types of microbial infections (Supplementary Material 10), organ failure (Supplementary Material 11). A matrix diagram of missing data is illustrated in the Data Profiling report (Supplementary Material 12). The percentage of missing values of partial thromboplastin time (0.47%), platelet (0.57%), aspartate aminotransferase (0.66%), alanine aminotransferase (0.47%), resprate (0.85%), heartrate (0.85%), blood urea nitrogen (1.14%), dysbp (1.14%), diasbp (1.23%), tempc (1.52%), glucose_min (1.42%), albumin (5.02%), lactate (3.6%), and hemoglobin (3.03%) were <6%. To facilitate statistical analysis, missing individual values were substituted with their mean values.

### Statistical Analysis

The Shapiro–Wilk test for the sample distribution was used. Continuous variables with normal distribution were expressed as the mean ± standard deviation (SD), and continuous non-normal distributed variables were expressed as the median (interquartile range, IQR), categorical variables were expressed as frequency and percentage, as appropriate. A non-parametric test (Mann–Whitney *U* test or Kruskal–Wallis test) was applied for data with non-normal distribution or heterogeneity of variances. Pearson Chi-squared test was applied to categorize variables.

Patients were randomly assigned to either the training cohort (80%) or the validation cohort (20%). The selection of predictive features of the nomogram used the least absolute shrinkage and selection operator (Lasso) regression model (Sauerbrei et al., 2007; Sun et al., 2013; Wang et al., 2020). A multivariate COX regression analysis was performed on the selected variables, and a nomogram was constructed based on the results of the multivariate COX regression analysis (*P* < 0.05). We applied a bootstrapped resample with 1,000 iterations to verify the accuracy of the nomogram. The C-index was employed as an indicator to determine the discrimination ability of the nomogram through receiver operating characteristic (ROC) curve analysis and area under the curve (AUC). The calibration was performed by plotting the calibration curve to analyze the association between the observed incidence and the predicted probability. We evaluated the clinical usefulness and net benefit of the new predictive models by using decision curve analysis (DCA).

Statistical analysis was conducted with R software (version 3.4.3). Statistical significance was defined as *p* < 0.05.

## Results

### Demographic Baseline Characteristics

**FIGURE 1:**
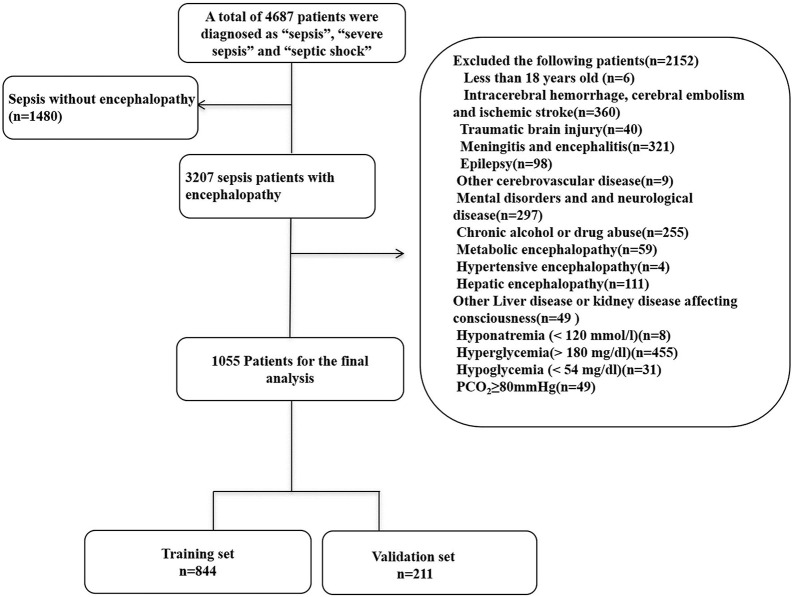
Flow chart of the enrolled patients. MIMIC-III, Medical Information Mart for Intensive Care III.

1,055 patients with SAE were identified from the MIMIC database after applying the inclusion and exclusion criteria. We randomly assigned 80% and 20% of the patients to the training (*n* = 844) and validation (*n* = 211) cohorts. The recruitment process is illustrated in Figure 1.

Table 1 shows the patient characteristics in the primary and validation cohorts. SAE patients who were older, had urinary tract infection or yeast infection were more likely to die. Circulatory failure was more common in non-survivors [Heartrate, 112 (96–132) vs. 109 (94–125), *P* = 0.008; Dysbp, 77 (64.8–86) vs. 85 (77–94), *P* < 0.001; Diasbp, 35 (26–41) vs. 39 (33–47), *P* < 0.001; Lactate, 2.3 (1.6–4.4) vs. 1.8 (1.2–2.7), *P* < 0.001] than survivors. Patients in the non-survival group had worse liver function [Alanine aminotransferase, 33 (17–84) vs. 24 (14–51.3), *P* = 0.001; Aspartate aminotransferase, 49 (25–139) vs. 31.5 (20–64), *P* < 0.001; Albumin, 2.8 (2.2–3.0) vs. 2.9 (2.5–3.3), *P* < 0.001], worse renal function [Creatinine, 1.1 (0.8–1.9) vs. 1.5 (0.9–2.6), *P* < 0.001; Blood urea nitrogen, 24 (16–41) vs. 33 (20–50.3), *P* < 0.001], and worse coagulation [Partial time, 14.3 (13.0–16.4) vs. 15.5 (13.8–19.6), *P* < 0.001; Partial thromboplastin time, 30.5 (26.9–36.9) vs. 36.7 (30.2–49.6), *P* < 0.001; INR 1.3 (1.1–1.5) vs. 1.5 (1.2–2.0), *P* < 0.001], and more serious infections [White blood cell count, 10.7 (7.2–15.3) vs. 11.9 (7.9–16.9), *P* = 0.061]. Patients in the non-survival group also had a higher incidence of anemia (53.2 vs. 67.8%). Non-surviving patients have more severe disease [SOFA 6.0 (9.0–12.0) vs. 6.0 (4.0–8.0), *p* < 0.001; qSOFA 2.0 (2.0–3.0) vs. 2.0 (2.0–3.0) *p* < 0.001; SAPS II 56 (45–72) vs. 42 (32–51), *p* < 0.001].

**TABLE 1:** Characteristics of patients in the primary and validation cohorts.

	Primary cohort			Validation cohort		
	Survive group	Non-survival group	*P*	Survive group	Non-survival group	*P*
	*n* = 586	*n* = 258		*n* = 147	*n* = 64	
Age, median	72 (59.0–82.4)	76.7 (64–84.4)	0.003	70.0 (56.8–81.7)	75.2 (65.2–83.7)	0.068
**Sex n (%)**						
Female	299 (51.0)	118 (45.7)	0.721	63 (42.9)	33 (51.6)	0.243
Male	287 (49.0)	140 (54.3)		84 (57.1)	31 (48.4)	
**Admission_type (%)**						
Emergency	535 (92.0)	248 (91.0)	0.043	136 (92.5)	59 (92.2)	0.014
Elective	38 (6.1)	8 (7.2)		9 (6.1)	4 (6.3)	
Urgent	13 (1.9)	2 (1.9)		2 (1.4)	1 (1.5)	
**Comorbidity, n (%)**						
Hypertension	365 (62.3)	146 (56.6)	<0.001	91 (61.9)	40 (62.5)	0.935
Diabetes	179 (30.5)	88 (34.1)	<0.001	41 (27.9)	19 (29.7)	0.790
Cardiovascular diseases	388 (66.2)	176 (68.2)	0.569	93 (63.3)	47 (73.4)	0.151
Chronic pulmonary disease	122 (20.8)	57 (22.1)	0.677	25 (17.0)	14 (21.9)	0.402
Liver disease	51 (8.7)	27 (10.5)	0.415	17 (11.6)	11 (17.2)	0.268
Anemias	312 (53.2)	175 (67.8)	<0.001	80 (54.4)	49 (76.6)	0.002
**Infection site, n (%)**						
Lung	235 (40.1)	98 (38.0)	0.562	66 (44.9)	27 (42.2)	0.715
Intestinal	108 (18.4)	41 (15.9)	0.373	30 (20.4)	11 (17.2)	0.587
Urinary system	238 (40.6)	61 (23.6)	<0.001	58 (39.5)	16 (25)	0.043
Catheter related	98 (16.7)	29 (11.2)	0.040	25 (17.0)	7 (10.9)	0.259
Skin and soft tissue	81 (13.8)	31 (12.0)	0.476	19 (12.9)	11 (17.2)	0.415
Abdominal cavity	135 (23.0)	60 (23.3)	0.945	31 (21.1)	17 (26.6)	0.383
**Microorganisms, n (%)**						
*Escherichia coli*	122 (20.8)	31 (12.0)	0.002	32 (21.8)	11 (17.2)	0.448
*Klebsiella oxytoca*	8 (1.4)	6 (2.3)	0.314	7 (4.8)	0 (0)	0.076
*Acinetobacter baumannii*	10 (1.7)	4 (1.6)	0.870	2 (1.4)	2 (3.1)	0.388
*Enterobacter*	28 (4.8)	7 (2.7)	0.166	1 (0.68)	2 (3.1)	0.168
*Staphylococcus aureus* coag	137 (23.4)	71 (27.5)	0.198	38 (25.9)	14 (21.9)	0.538
*Pseudomonas aeruginosa*	78 (13.3)	22 (8.5)	0.048	16 (10.9)	10 (15.6)	0.336
*Enterococcus* sp.	130 (22.2)	45 (17.4)	0.034	43 (29.3)	10 (15.6)	0.036
*Streptococcus*	21 (3.6)	7 (2.7)	0.515	7 (4.8)	1 (1.5)	0.263
*Candida albicans*	20 (3.4)	13 (5.0)	0.262	11 (7.5)	3 (4.7)	0.453
Yeast	178 (30.4)	101 (39.1)	0.013	64 (43.5)	32 (5.0)	0.386
*Aspergillus fumigatus*	14 (2.4)	6 (2.3)	0.955	0 (0)	1 (1.5)	0.129
Positive for methicillin resistant *Staphylococcus aureus*	41 (7.0)	8 (3.1)	0.026	8 (5.4)	4 (6.3)	0.816
*Staphylococcus* coagulase negative	140 (23.9)	51 (19.8)	0.187	32 (21.8)	10 (15.6)	0.304
Virus	3 (0.51)	3 (1.2)	0.300	4 (2.7)	0 (0)	0.183
**Vital signs, median**						
Heartrate (bpm)	109 (94–125)	112 (96–132)	0.008	111.9 ± 22.7	117.4 ± 22.7	0.055
Dysbp (mmHg)	85 (77–94)	77 (64.8–86)	<0.001	83.5 (75–94.8)	78 (62–89)	0.009
Diasbp (mmHg)	39 (33–47)	35 (26–41)	<0.001	39.5 ± 10.9	36.4 ± 13.8	0.047
Resprate (bpm)	28 (24–32)	29 (25–35)	0.021	29 (25–33.5)	29 (26–34)	0.283
Tempc (°C)	37.6 (36.9–38.3)	37.4 (36.7–38.0)	0.002	37.7 (37.0–38.3)	37.3 (36.7–38.2)	0.042
**Laboratory parameters**						
Lactate (mmol/L)	1.8 (1.2–2.7)	2.3 (1.6–4.4)	<0.001	1.9 (1.3–2.6)	2.3 (1.3–4.2)	0.088
PCO_2_ (mmHg)	40 (36–42)	40 (32–45)	0.874	40 (36–44)	40 (35–44)	0.592
PO_2_ (mmHg)	92 (90–95)	91 (83.8–93)	<0.001	93 (89.5–95)	89 (80–95)	0.018
PH	7.34 (7.34–7.39)	7.34 (7.28–7.41)	0.142	7.34 (7.32–7.42)	7.34 (7.25–7.40)	0.229
Glucose_min (mg/dL)	101 (85–120)	99 (75–122)	0.053	104.1 ± 30.5	99.0 ± 31.4	0.378
Glucose_max (mg/dL)	122 (156–200)	165 (139–205.3)	0.116	147 (124–202.5)	183.5 (147.5–234.5)	0.001
Creatinine (mg/dL)	1.1 (0.8–1.9)	1.5 (0.9–2.6)	<0.001	1.0 (0.7–1.4)	1.4 (0.7–2.5)	0.050
Blood urea nitrogen (mg/dL)	24 (16–41)	33 (20–50.3)	<0.001	22 (13–36)	29.0 (18.3–54.8)	0.005
Alanine aminotransferase (IU/L)	24 (14–51.3)	33 (17–84)	0.001	26.5 (15–43.5)	31 (15.8–49.3)	0.608
Aspartate aminotransferase (IU/L)	31.5 (20–64)	49 (25–139)	<0.001	31 (18–51)	35 (23.8–70.3)	0.090
Albumin (g/dL)	2.9 (2.5–3.3)	2.8 (2.2–3.0)	<0.001	2.9 (2.5–3.2)	2.8 (2.2–3.3)	0.532
Hemoglobin (g/dL)	9.6 (8.5–10.7)	9.7 (8.6–10.8)	0.728	9.7 (8.6–11.1)	9.6 (8.9–11.3)	0.630
Platelet (K/uL)	182.5 (122–272.3)	171 (110.8–246)	0.082	174.0 (106–246)	181.0 (118–271)	0.269
Potassium (mEq/L)	4.0 (3.6–4.4)	4.1 (3.8–4.6)	0.005	4.0 (3.7–4.2)	4.3 (3.8–4.5)	0.010
Sodium (mEq/L)	139 (136–142)	139 (134–142)	0.027	139 (136–142)	138 (135–141)	0.200
Partial time (s)	14.3 (13.0–16.4)	15.5 (13.8–19.6)	<0.001	14.1 (13.0–16.3)	14.7 (13.4–18.2)	0.080
Partial hromboplastin time	30.5 (26.9–36.9)	36.7 (30.2–49.6)	<0.001	29.7 (26.1–36.3)	33.5 (28.9–40.3)	0.004
INR	1.3 (1.1–1.5)	1.5 (1.2–2.0)	<0.001	1.3 (1.1–1.5)	1.4 (1.2–1.9)	0.030
White blood cell count (K/uL)	10.7 (7.2–15.3)	11.9 (7.9–16.9)	0.061	10.2 (6.6–14.3)	11.8 (8.8–15.3)	0.029
Lymphocyte (%)	9.8 (5.4–16.4)	8.0 (5.0–14.0)	0.017	11.2 (6.3–17.6)	10.3 (5.1–16.7)	0.557
Neutrophil (%)	80.1 (70.0–87.5)	80.8 (71.2–88)	0.426	80.0 (70.1–87.8)	82.3 (69.8–89.0)	0.445
Monocytes (%)	4.0 (2.5–6.0)	4.0 (2.0–6.4)	0.776	4.1 (2.7–6.0)	3.9 (2.5–5.2)	0.393
Eosinophils (%)	0.6 (0.0–2.0)	0.2 (0.0–1.2)	0.001	0.55 (0.08–1.4)	0.3 (0.0–1.4)	0.423
**Severe Score**						
SOFA	6.0 (4.0–8.0)	6.0 (9.0–12.0)	<0.001	5.0 (3.0–8.0)	9.5 (6.0–12.8)	<0.001
qSOFA	2.0 (2.0–3.0)	2.0 (2.0–3.0)	<0.001	2.0 (2.0–3.0)	2.5 (2.0–3.0)	0.003
SAPSII	42 (32–51)	56 (45–72)	<0.001	41 (32–50)	58 (45–70)	<0.001
GCS	14 (11–14)	13 (8–14)	<0.001	14 (10–14)	13 (14–8.25)	0.056

*SAPSII, patients’ simplified acute physiology score; SOFA, sequential organ failure assessment; qSOFA, quick sequential organ failure assessment; GCS, Glasgow coma scale; INR: international normalized ratio.*

### Patient Outcomes

Table 2 shows the outcomes for the survival group and non-survival group. Among non-survivors, there was a higher incidence of multiple organ failure including respiratory failure (63.6 vs. 32.9%), renal failure (69.4 vs. 57.3%), hepatic failure (10.5 vs. 3.3%), cardiovascular failure (58.1 vs. 8.5%), and hematological failure (26.7 vs. 21.2%). This led to a higher rate of mechanical ventilation (52.7 vs. 35.2%) and renal replacement therapy (12.0 vs. 2.0%) among non-survivors.

**TABLE 2:** Patients’ Outcome in the primary and validation cohorts.

	Primary cohort			Validation cohort		
	Survive group	Non-survival group	*P*	Survive group	Non-survival group	*P*
	*n* = 586	*n* = 258		*n* = 147	*n* = 64	
Mechanical ventilation, n (%)	206 (35.2)	136 (52.7)	<0.001	60 (40.8)	36 (56.3)	0.038
Renal replacement therapy, n (%)	12 (2.0)	31 (12.0)	<0.001	9 (6.1)	5 (7.8)	0.650
**Organ failure (%)**						
Respiratory	193 (32.9)	164 (63.6)	<0.001	58 (39.5)	45 (70.3)	<0.001
Cardiovascular	50 (8.5)	150 (58.1)	<0.001	59 (40.1)	40 (62.5)	0.003
Renal	336 (57.3)	179 (69.4)	<0.001	78 (53.1)	44 (68.8)	0.034
Hepatic	29 (3.3)	27 (10.5)	<0.001	6 (4.1)	7 (10.9)	0.057
Hematologic	124 (21.2)	69 (26.7)	<0.001	31 (21.1)	25 (39.1)	0.007
ICU stay time, days	2.8 (1.7–5.6)	3.4 (1.8–8.7)	<0.001	3.0 (1.8–6.8)	2.7 (1.1–7.2)	0.234

*ICU, intensive care unit.*

### Feature Selection

**FIGURE 2:**
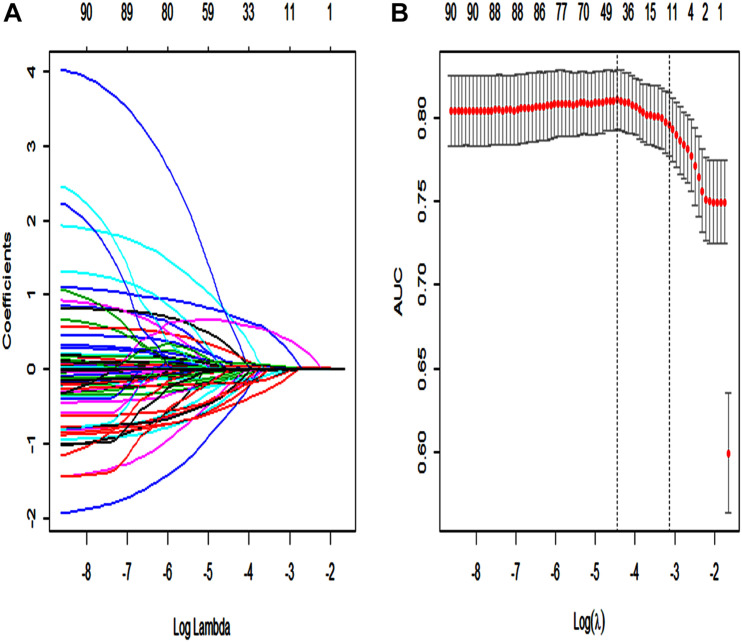
Texture feature selection using the least absolute shrinkage and selection operator (LASSO) binary logistic regression model. **(A)** Each curve in the figure represents the change trajectory of each independent variable coefficient. The ordinate is the value of the coefficient, the lower abscissa is log(λ), and the upper abscissa is the number of non-zero coefficients in the model at this time. **(B)** 10-fold cross-cross validation fitting and then select the model, and at the same time have a more accurate estimate of the performance of the model. For each λ value, around the mean value of the target parameter shown by the red dot, we can get a confidence interval for the target parameter. The two dashed lines indicate two special λ values:c (cvfit$lambda.min, cvfit$lambda.1se). The area under the receiver operating characteristic (AUC) curve was plotted vs. log(λ).

Using the LASSO regression model, among the non-survivors of SAE, we identified 89 features which reduced to 13 potential predictors. They include SAPS II, renal replacement therapy, temperature, SpO_2_, albumin, INR, lactate, respiratory failure, urinary tract infection, anemia, systolic blood pressure (sysbp), partial thromboplastin time (Supplementary Material 13: Data supplement) (Figures 2A,B).

### Multivariate Cox Regression

Furthermore, we performed a univariate, and multivariate cox regression analysis of these 13 potential predictors, sex, and admission type. According to our results, SAPS II, renal replacement therapy, temperature, SpO_2_, albumin, international normalized ratio (INR), lactate, and respiratory failure were independent prognostic factors for SAE patients (*p* < 0.01 or *p* < 0.05) (Table 3).

**TABLE 3:** Multivariate COX analysis of risk factors to hospital mortality.

	Multivariate analysis			
	RR	95.0% CI		*p*-values
		Lower	Upper	
Sex *n* (%)	1.018	0.857	1.207	0.842
Female				
Male				
Admission_type (%)				
Emergency	1.000			
Elective	1.209	0.546	2.676	0.639
Urgent	1.924	0.950	3.897	0.069
Urinary tract Infection (%)	1.022	0.849	1.230	0.817
Anemias (%)	0.363	0.912	1.286	0.363
SAPSII	1.013	1.008	1.018	<0.001
Renal replacement therapy, *n* (%)	2.282	1.598	3.258	<0.001
Sysbp (mmHg)	0.998	0.992	1.004	0.462
Tempc (°C)	0.776	0.703	0.857	<0.001
SpO_2_ (mmHg)	0.989	0.980	0.997	0.012
Albumin (g/dL)	1.182	1.040	1.343	0.011
INR	1.140	1.064	1.222	<0.001
Partial time (s)	0.997	0.987	1.008	0.645
lactate (mmol/l)	1.070	1.039	1.103	<0.001
Respiratory failure (%)	1.847	1.540	2.215	<0.001

*SAPSII, patients’ simplified acute physiology score; INR, international normalized ratio.*

### Predictive Nomogram Development

**FIGURE 3:**
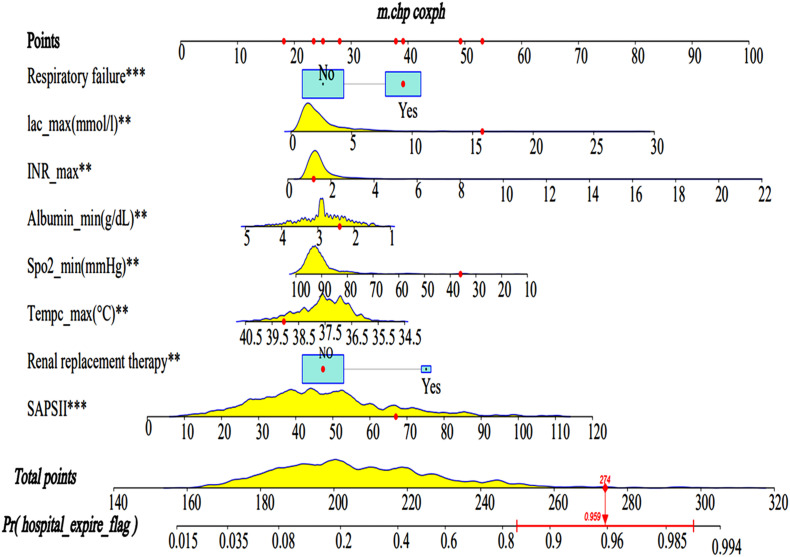
Nomograms for the prediction of the hospital mortality of SAE patients. The line segment corresponding to each variable is marked with a scale, which represents the value range of the variable, and the length of the line segment reflects the contribution of the factor to the hospital mortality of SAE patients. The Point in the figure represents the individual score corresponding to each variable under different values, and the total score, namely Total Point, represents the total score of the sum of the corresponding individual scores after all the variables are valued. SAPS II, simplified acute physiology score; Lac, lactate. ** < 0.05, *** < 0.01.

A Lasso regression model and multivariate cox regression analysis identified SAPS II, renal replacement therapy, temperature, SpO_2_, albumin, INR, lactate, and respiratory failure as independent prognostic factors for SAE patients in the training cohort. These factors can be used to predict the hospital mortality of patients with SAE (Table 3), which was presented as the visualization nomogram (Figure 3). The hazard ratio values of these risk factors were established and scored for each level of prognostication. By adding up the scores associated with each variable to assess the hospital mortality of SAE patients.

### Discrimination and Calibration

**FIGURE 4:**
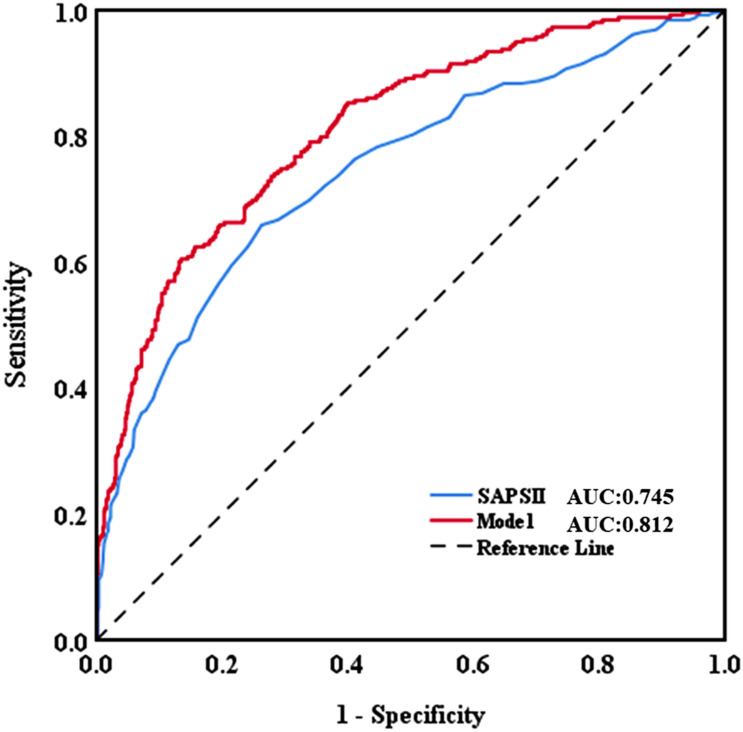
Discriminatory accuracy for predicting the incidence of SAE assessed by receiver operator characteristics (ROC) analysis calculating area under the curve (AUC). SAPS II, simplified acute physiology score.

**FIGURE 5:**
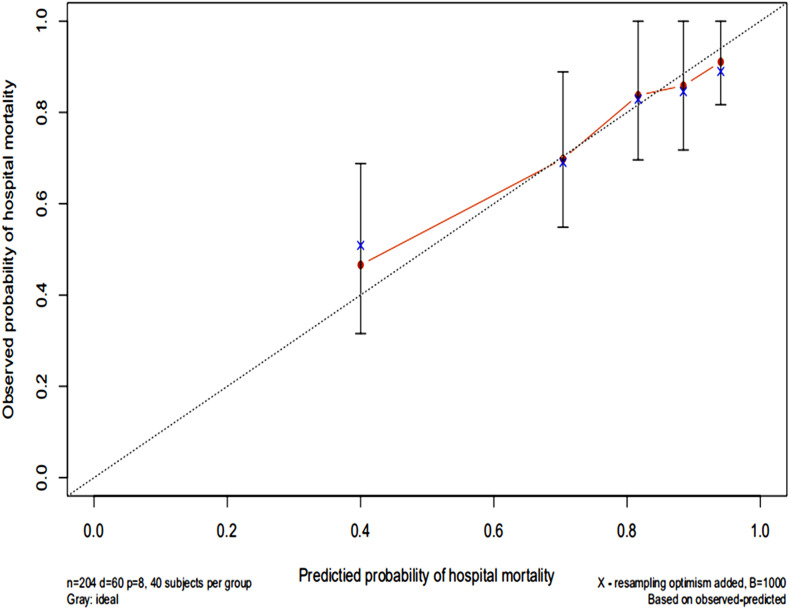
Calibration curves of a nomogram estimating the hospital mortality of SAE patients. The x-axis represents the predicted risk of hospital death in patients with SAE. The y-axis represents the actual risk of hospital death in patients with SAE. The dotted line represents the perfect prediction of the ideal model. The closer the red solid line is to the dotted line, the better the performance of this nomogram.

The AUC for the hospital mortality prediction nomogram was 0.812 (95% CI, 0.780–0.843) in the training cohort, which is greater than the SAPS II score of 0.745 (95% CI, 0.708–0.783) (Figure 4). The predictive accuracy of the nomogram was shown with a sensitivity of 0.601 and a specificity of 0.867. Our study employed the bootstrap resampling method for internal validation of the model. The calibration plot of hospital mortality of SAE patients revealed good agreement between the observed and predicted values (Figure 5).

### Clinical Utility

**FIGURE 6:**
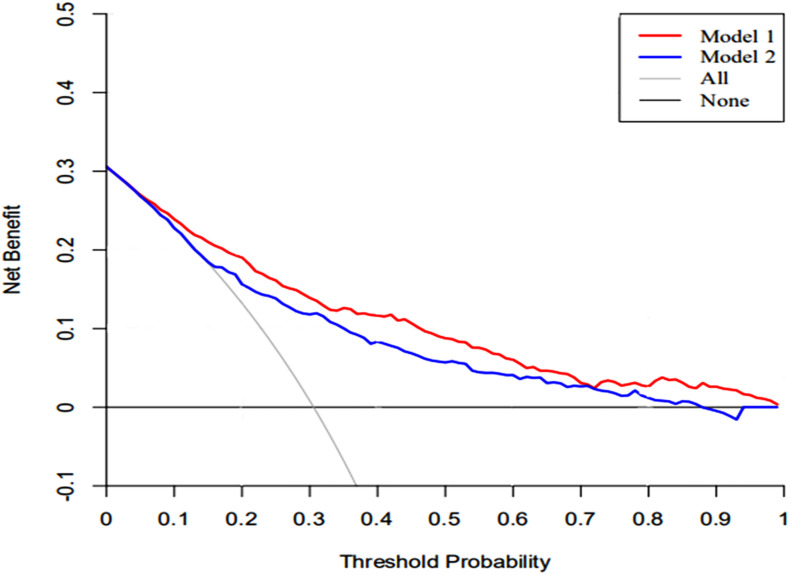
The DCA curve of medical intervention in patients with the nomogram and SAPS II. Model 1: nomogram; Model 2: SAPS II. Solid line: The patient does not apply the nomogram, and the net income is zero; Gray line: All patients used the nomogram. The further the red solid line and blue solid line are from the dotted line, the greater the clinical application value.

The DCA of the nomograms and SAPS II for the hospital mortality of patients with SAE are illustrated in Figure 6. The results showed that the nomogram provided a greater net benefit in predicting hospital mortality compared to that of SAPS II.

## Discussion

In our study, patients with SAE have a hospitalized mortality rate of 30.5%. Intestinal infections and microbial infections were not found to be related to the prognosis of SAE patients. We identified independent factors for the prognosis of SAE patients, which included SAPS II, renal replacement therapy, albumin, INR, lactate, temperature, SpO_2_, and respiratory failure. We further developed and validated a comprehensive visual nomogram to predict the prognosis of SAE patients. The nomogram showed a high degree of validity, discrimination, and clinical utility.

Microorganisms in the body are related to many diseases. Li-Hong Peng and Lihong Peng et al. (2018, 2020a) established a model to predict the association of microorganisms with various diseases through microorganisms, and the model showed excellent performance. Probiotics can change the types of intestinal microflora and affect patients’ mood and memory function (Bagga et al., 2018). Xia et al. (2018) found that probiotics can improve the cognitive function of patients with hepatic encephalopathy. Wei et al. (2020) found that Enterobacteriaceae can improve patients’ mild cognitive impairment. Although the study of Li et al. (2018) has proved that the intestinal flora could affect SAE through the vagus nerve. Unfortunately, our study found that intestinal infections and microbes have nothing to do with the prognosis of SAE patients. It may require further experimental study in the future.

The SAPS II was developed from a European/North American study. Patients included in that study were from medical and surgical wards, as well as ICUs, in ten European and two North American countries. The authors showed that SAPS II demonstrated a high level of predictivity on the death of hospitalized patients (Le Gall et al., 1993). Although later studies have suggested better predictive tools than SAPS II (Norrie, 2015), our cohort study showed that the SAPS II score of non-surviving patients was significantly higher than that of patients in the survival group, which further supports the accuracy of SAPS II as an independent predictive factor for hospital mortality in SAE patients.

Our cohort study demonstrated that the incidence of renal replacement therapy in the non-survival group was significantly higher than that in the survival group. After LASSO and multivariate Cox regression analyses, it was found to be an independent risk factor for the death of SAE. However, the use of renal replacement therapy cannot be assumed to be an independent factor for death. Patients who were given renal replacement therapy were more likely to be severely ill with worse kidney function, more serious infection, and a higher incidence of multiple organ dysfunction, and internal environmental disorders (Palevsky, 2008; Bagshaw and Wald, 2018; Tandukar and Palevsky, 2018). This, in turn, leads to a higher mortality rate. Our cohort study also showed that SAE patients with respiratory failure, worse coagulation function, and lower albumin levels were more likely to die. The mechanism of multiple organ dysfunction in patients with SAE is consistent with sepsis patients, and it may be attributed to the immune response to sepsis (Nolt et al., 2018); circulatory abnormalities (De Backer et al., 2013; Finfer et al., 2013), organ ischemia; hypoxia endothelial permeability increases (Kopterides et al., 2011; Opal and van der Poll, 2015); cell death (Pinheiro da Silva and Nizet, 2009); and mitochondrial dysfunction (Yang et al., 2015; Sun et al., 2019). We should promptly correct the respiratory failure, give component blood transfusions, correct coagulation function, supplement albumin, and reduce the mortality of SAE patients.

Lactate is a vital laboratory indicator that affects the prognosis of patients with sepsis. It is widely known, the higher the lactate level, the worse the patient’s prognosis (Suetrong and Walley, 2016; Liu et al., 2019). Serum lactate is also an independent risk factor for the prognosis of SAE patients in our cohort study. In patients with septic shock, fluid resuscitation guided by monitoring the serum lactate is still the most effective method for reducing the mortality of septic shock (Hernández et al., 2019). Serum lactate is used to evaluate disease severity, guide treatment plan, and predict patient prognosis (Suetrong and Walley, 2016). Lower serum lactate levels are associated with reduced patient mortality (Puskarich et al., 2013; Vincent et al., 2016). Therefore, serum lactate is an important indicator for evaluating the prognosis of patients with sepsis and SAE. The results of previous studies further support our conclusion. Patients with lactate acidosis and hyperlactic acidosis, we should timely rehydration and other treatments to reduce lactate levels and improve the survival rate of SAE patients.

There is currently a lack of effective tools for predicting hospital mortality in SAE patients. By exploring the clinical indicators for evaluating the prognosis of SAE patients, through Lasso and Cox regression analysis, eight potential predictors, including SAPS II, renal replacement therapy, albumin, INR, lactate, body temperature, SpO_2_, respiratory failure were identified and used to establish a comprehensive visual nomogram for predicting hospital mortality of SAE patient. The nomogram demonstrated excellent discrimination (AUC, 0.812; 95%CI: 0.780–0.843) that was better than SAPS II (AUC, 0.745; 95%CI: 0.708–0.783) in the primary cohort. The validation cohort is used to verify the calibration function of the nomogram and has good consistency with the model (Figure 5). In terms of clinical application, the net benefit of patients using nomogram is better than that of SAPS II (Figure 6), and the nomogram shows good performance in predicting hospital mortality of SAE patients. For the evaluation of nomograms, in addition to the above-mentioned AUC value and other methods, some new methods may be needed to evaluate in the future (Zhou et al., 2019; Liu F. et al., 2020).

Several limitations must be acknowledged. Firstly, our study is retrospective based on the MIMIC database, which has its inherent limitations. For instance, our study identified septic patients using the definition from the ICD-9 diagnostic code, which may be different from the Sepsis-3 definition. However, this small discrepancy does not deny the clinical application value of our study. Although our nomogram has excellent performance, our data is older and we need new data to verify in the future. Secondly, we included ICU patients for analysis, which enhanced the heterogeneity of the study population, and thus our results may not be suitable for patients outside the ICU. Third, there are a lot of more widely used methods in feature selection and classification than Lasso, such as elastic net, random forest, and deep neural network (Huang et al., 2017; He et al.,2020a,b; Liang et al., 2020; Liu C. et al., 2020; Yang J. et al., 2020). Model development only uses the general linear regression method, fusing various biological information by multi-information fusion (Peng et al., 2017), bipartite local model (Peng et al., 2020b), and the KATZ method (Zhou et al., 2020) should be further studied in the future. We will apply these methods to further improve the performance of our model. Finally, the Model establishment was only verified internally, and further external verification is required in the future to illustrate its extrapolation.

## Conclusion

A nomogram was established for predicting hospital mortality of SAE patients, which was accurate and clinically useful. The nomogram also performed better than the SAPS II with a higher net benefit.

## Data Availability Statement

The original contributions presented in the study are included in the article/Supplementary Material, further inquiries can be directed to the corresponding author/s.

## Ethics Statement

The studies involving human participants were reviewed and approved by the establishment of the database was approved by the Massachusetts Institute of Technology (Cambridge, MA, United States) and the Institutional Review Boards of Beth Israel Deaconess Medical Center (Boston, MA, United States). Written informed consent for participation was not required for this study in accordance with the national legislation and the institutional requirements.

## Author Contributions

LZ, YL, XS, and YL developed this manuscript central ideas. YW and ZG collected the data regarding the manuscript. LZ wrote the first draft of the manuscript. YL and XS revised the manuscript, worked on the English, and made the final version of the manuscript. All authors read and approved the final manuscript.

## Conflict of Interest

The authors declare that the research was conducted in the absence of any commercial or financial relationships that could be construed as a potential conflict of interest.

## Publisher’s Note

All claims expressed in this article are solely those of the authors and do not necessarily represent those of their affiliated organizations, or those of the publisher, the editors and the reviewers. Any product that may be evaluated in this article, or claim that may be made by its manufacturer, is not guaranteed or endorsed by the publisher.

## Supplementary Material

Supplementary Material 1 Exclusion of patients with traumatic injury from the MIMIC III database according to ICD-9 codes.

Supplementary Material 2 Exclusion of patients with intracerebral hemorrhage, cerebral embolism, and ischemic stroke disease from the MIMIC III database according to ICD-9 codes.

Supplementary Material 3 Exclusion of patients with meningitis and encephalitis from the MIMIC III database according to ICD-9 codes.

Supplementary Material 4 Exclusion of patients with Epilepsy disease from the MIMIC III database according to ICD-9 codes.

Supplementary Material 5 Exclusion of patients with other cerebrovascular disease from the MIMIC III database according to ICD-9 codes.

Supplementary Material 6 Exclusion of patients with mental disorders and neurological disease from the MIMIC III database according to ICD-9 codes.

Supplementary Material 7 Exclusion of patients with alcohol intoxication/abuse or drug abuse from the MIMIC III database according to ICD-9 codes.

Supplementary Material 8 Exclusion of patients with metabolic encephalopathy, hepatic encephalopathy, hypertensive encephalopathy, diabetes with coma, disorders of the urea cycle, hypernatremia from the MIMIC III database according to ICD-9 codes.

Supplementary Material 9 Type of disease and ICD-9 codes.

Supplementary Material 10 Infection site and ICD-9 codes.

Supplementary Material 11 Organ failure was defined by a combination of ICD-9-CM and Current Procedural Terminology (CPT) codes.

Supplementary Material 12 Data profiling report.

Supplementary Material 13 Data supplement, potential predictors by LASSO logistic regression model.

## Abbreviations

SAE: sepsis-associated encephalopathy
SAPS II: simplified acute physiology score
SOFA: sequential organ failure assessment
qSOFA: quick sequential organ failure assessment
GCS: Glasgow coma scale
ICU: intensive care unit
MIMIC-III: Medical Information Mart for Intensive Care III
ICD-9: International Classification of Diseases Ninth Revision
ROC: receiver operating characteristic curve
INR: international normalized ratio.
