# Corrigendum: Development and Validation of a Nomogram for the Prediction of Hospital Mortality of Patients With Encephalopathy Caused by Microbial Infection: A Retrospective Cohort Study

**DOI:** 10.3389/fmicb.2021.773499

**Published:** 2021-11-03

**Authors:** Lina Zhao, Yun Li, Yunying Wang, Qian Gao, Zengzheng Ge, Xibo Sun, Yi Li

**Affiliations:** ^1^Emergency Department, State Key Laboratory of Complex Severe and Rare Diseases, Peking Union Medical College Hospital, Peking Union Medical College, Chinese Academy of Medical Sciences, Beijing, China; ^2^Department of Critical Care Medicine, Chifeng Municipal Hospital, Chifeng Clinical Medical College of Inner Mongolia Medical University, Chifeng, China; ^3^Department of Anesthesiology, Chifeng Municipal Hospital, Chifeng Clinical Medical College of Inner Mongolia Medical University, Chifeng, China; ^4^Department of Neurology, Yidu Central Hospital Affiliated to Weifang Medical University, Weifang, China

**Keywords:** sepsis associated encephalopathy, prognosis, hospital mortality, nomogram, microbial infection

In the original article, there was a mistake in Figure 1 as published. Patients with sepsis without encephalopathy should be 1480. We wrote this as 1408 by error. Therefore 3279 patients for the final analysis should be modified to 3207. In the excluded patients, all excluded patients add up to 2152, not 3560. Therefore, there are three numbers in Figure 1 that need to be changed. (1) “sepsis without encephalopathy (n=1408)” changed to “sepsis without encephalopathy (n=1480).” (2) “3279 patients for the final analysis” changed to “3207 patients for the final analysis.” (3) “Exclude the following patients (n=3560)” changed to “Exclude the following patients (n=2152).” The corrected Figure 1 appears below.

**Figure 1:**
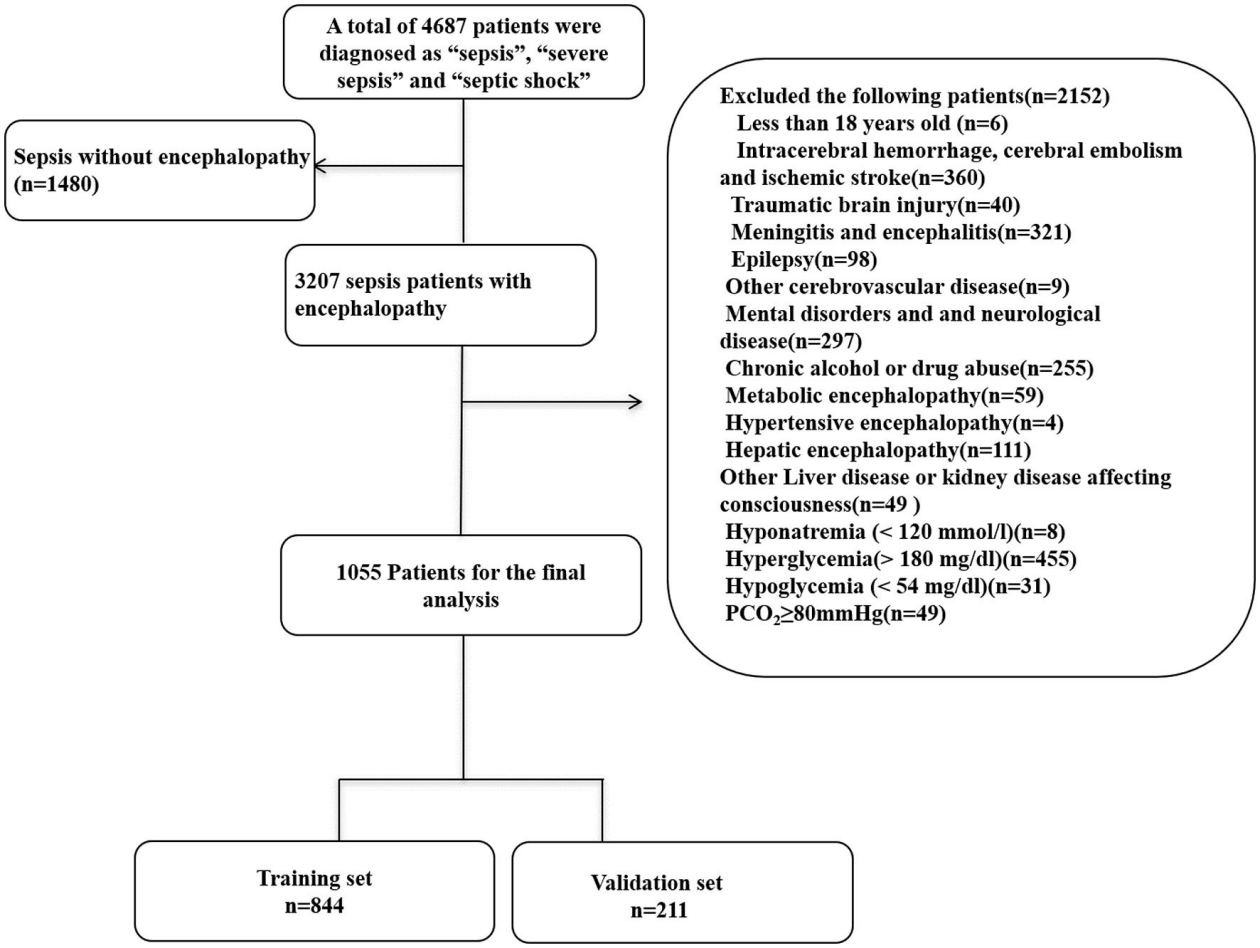
Flow chart of the enrolled patients. MIMIC-III, Medical Information Mart for Intensive Care III.

We apologize for this error and state that this does not change the scientific conclusions of the article in any way. The original article has been updated.

## Publisher's Note

All claims expressed in this article are solely those of the authors and do not necessarily represent those of their affiliated organizations, or those of the publisher, the editors and the reviewers. Any product that may be evaluated in this article, or claim that may be made by its manufacturer, is not guaranteed or endorsed by the publisher.

