# Supplementary material for: Development and Validation of a Nomogram for the Prediction of Hospital Mortality of Patients With Encephalopathy Caused by Microbial Infection: A Retrospective Cohort Study
Source: Front Microbiol. 2021 Aug 19;12:737066. doi: 10.3389/fmicb.2021.737066 (PMC8417384; doi:10.3389/fmicb.2021.737066)
Supplement: Supplementary Material 1 — Exclusion of patients with traumatic injury from the MIMIC III database according to ICD-9 codes. [file Data_Sheet_1.zip › Supplementary Material 1.docx]

| **Supplementary material 1** Exclude patients with trauma of skull from the MIMIC III database according to ICD9-codes | | |
| --- | --- | --- |
| ICD9-code |  | Description |
| 80016 |  | Closed fracture of vault of skull with cerebral laceration and contusion, with loss of consciousness of unspecified duration |
| 80019 |  | Closed fracture of vault of skull with cerebral laceration and contusion, with concussion, unspecified |
| 80020 |  | Closed fracture of vault of skull with subarachnoid, subdural, and extradural hemorrhage, unspecified state of consciousness |
| 80021 |  | Closed fracture of vault of skull with subarachnoid, subdural, and extradural hemorrhage, with no loss of consciousness |
| 80022 |  | Closed fracture of vault of skull with subarachnoid, subdural, and extradural hemorrhage, with brief [less than one hour] loss of consciousness |
| 80023 |  | Closed fracture of vault of skull with subarachnoid, subdural, and extradural hemorrhage, with moderate [1-24 hours] loss of consciousness |
| 80024 |  | Closed fracture of vault of skull with subarachnoid, subdural, and extradural hemorrhage, with prolonged [more than 24 hours] loss of consciousness and return to pre-existing conscious level |
| 80025 |  | Closed fracture of vault of skull with subarachnoid, subdural, and extradural hemorrhage, with prolonged [more than 24 hours] loss of consciousness, without return to pre-existing conscious level |
| 80026 |  | Closed fracture of vault of skull with subarachnoid, subdural, and extradural hemorrhage, with loss of consciousness of unspecified duration |
| 80029 |  | Closed fracture of vault of skull with subarachnoid, subdural, and extradural hemorrhage, with concussion, unspecified |
| 80030 |  | Closed fracture of vault of skull with other and unspecified intracranial hemorrhage, unspecified state of consciousness |
| 80031 |  | Closed fracture of vault of skull with other and unspecified intracranial hemorrhage, with no loss of consciousness |
| 80032 |  | Closed fracture of vault of skull with other and unspecified intracranial hemorrhage, with brief [less than one hour] loss of consciousness |
| 80033 |  | Closed fracture of vault of skull with other and unspecified intracranial hemorrhage, with moderate [1-24 hours] loss of consciousness |
| 80034 |  | Closed fracture of vault of skull with other and unspecified intracranial hemorrhage, with prolonged [more than 24 hours] loss of consciousness and return to pre-existing conscious level |
| 80035 |  | Closed fracture of vault of skull with other and unspecified intracranial hemorrhage, with prolonged [more than 24 hours] loss of consciousness, without return to pre-existing conscious level |
| 80036 |  | Closed fracture of vault of skull with other and unspecified intracranial hemorrhage, with loss of consciousness of unspecified duration |
| 80129 |  | Closed fracture of base of skull with subarachnoid, subdural, and extradural hemorrhage, with concussion, unspecified |
| 80130 |  | Closed fracture of base of skull with other and unspecified intracranial hemorrhage, unspecified state of consciousness |
| 80131 |  | Closed fracture of base of skull with other and unspecified intracranial hemorrhage, with no loss of consciousness |
| 80132 |  | Closed fracture of base of skull with other and unspecified intracranial hemorrhage, with brief [less than one hour] loss of consciousness |
| 80133 |  | Closed fracture of base of skull with other and unspecified intracranial hemorrhage, with moderate [1-24 hours] loss of consciousness |
| 80134 |  | Closed fracture of base of skull with other and unspecified intracranial hemorrhage, with prolonged [more than 24 hours] loss of consciousness and return to pre-existing conscious level |
| 80135 |  | Closed fracture of base of skull with other and unspecified intracranial hemorrhage, with prolonged [more than 24 hours] loss of consciousness, without return to pre-existing conscious level |
| 80136 |  | Closed fracture of base of skull with other and unspecified intracranial hemorrhage, with loss of consciousness of unspecified duration |
| 80139 |  | Closed fracture of base of skull with other and unspecified intracranial hemorrhage, with concussion, unspecified |
| 80140 |  | Closed fracture of base of skull with intracranial injury of other and unspecified nature, unspecified state of consciousness |
| 80141 |  | Closed fracture of base of skull with intracranial injury of other and unspecified nature, with no loss of consciousness |
| 80142 |  | Closed fracture of base of skull with intracranial injury of other and unspecified nature, with brief [less than one hour] loss of consciousness |
| 80143 |  | Closed fracture of base of skull with intracranial injury of other and unspecified nature, with moderate [1-24 hours] loss of consciousness |
| 80144 |  | Closed fracture of base of skull with intracranial injury of other and unspecified nature, with prolonged [more than 24 hours) loss of consciousness and return to pre-existing conscious level |
| 80145 |  | Closed fracture of base of skull with intracranial injury of other and unspecified nature, with prolonged [more than 24 hours] loss of consciousness, without return to pre-existing conscious level |
| 80146 |  | Closed fracture of base of skull with intracranial injury of other and unspecified nature, with loss of consciousness of unspecified duration |
| 80149 |  | Closed fracture of base of skull with intracranial injury of other and unspecified nature, with concussion, unspecified |
| 80150 |  | Open fracture of base of skull without mention of intracranial injury, unspecified state of consciousness |
| 80151 |  | Open fracture of base of skull without mention of intracranial injury, with no loss of consciousness |
| 80152 |  | Open fracture of base of skull without mention of intracranial injury, with brief [less than one hour] loss of consciousness |
| 80153 |  | Open fracture of base of skull without mention of intracranial injury, with moderate [1-24 hours] loss of consciousness |
| 80154 |  | Open fracture of base of skull without mention of intracranial injury, with prolonged [more than 24 hours] loss of consciousness and return to pre-existing conscious level |
| 80155 |  | Open fracture of base of skull without mention of intracranial injury, with prolonged [more than 24 hours] loss of consciousness, without return to pre-existing conscious level |
| 80156 |  | Open fracture of base of skull without mention of intracranial injury, with loss of consciousness of unspecified duration |
| 80159 |  | Open fracture of base of skull without mention of intracranial injury, with concussion, unspecified |
| 80160 |  | Open fracture of base of skull with cerebral laceration and contusion, unspecified state of consciousness |
| 80161 |  | Open fracture of base of skull with cerebral laceration and contusion, with no loss of consciousness |
| 80162 |  | Open fracture of base of skull with cerebral laceration and contusion, with brief [less than one hour] loss of consciousness |
| 80163 |  | Open fracture of base of skull with cerebral laceration and contusion, with moderate [1-24 hours] loss of consciousness |
| 80164 |  | Open fracture of base of skull with cerebral laceration and contusion, with prolonged [more than 24 hours] loss of consciousness and return to pre-existing conscious level |
| 80165 |  | Open fracture of base of skull with cerebral laceration and contusion, with prolonged [more than 24 hours] loss of consciousness, without return to pre-existing conscious level |
| 80166 |  | Open fracture of base of skull with cerebral laceration and contusion, with loss of consciousness of unspecified duration |
| 80169 |  | Open fracture of base of skull with cerebral laceration and contusion, with concussion, unspecified |
| 80170 |  | Open fracture of base of skull with subarachnoid, subdural, and extradural hemorrhage, unspecified state of consciousness |
| 80171 |  | Open fracture of base of skull with subarachnoid, subdural, and extradural hemorrhage, with no loss of consciousness |
| 80172 |  | Open fracture of base of skull with subarachnoid, subdural, and extradural hemorrhage, with brief [less than one hour] loss of consciousness |
| 80173 |  | Open fracture of base of skull with subarachnoid, subdural, and extradural hemorrhage, with moderate [1-24 hours] loss of consciousness |
| 85154 |  | Cerebellar or brain stem contusion with open intracranial wound, with prolonged [more than 24 hours] loss of consciousness and return |
| 85155 |  | Cerebellar or brain stem contusion with open intracranial wound, with prolonged [more than 24 hours] loss of consciousness without return to pre-existing conscious level |
| 85156 |  | Cerebellar or brain stem contusion with open intracranial wound, with loss of consciousness of unspecified duration |
| 85159 |  | Cerebellar or brain stem contusion with open intracranial wound, with concussion, unspecified |
| 85160 |  | Cerebellar or brain stem laceration without mention of open intracranial wound, unspecified state of consciousness |
| 85161 |  | Cerebellar or brain stem laceration without mention of open intracranial wound, with no loss of consciousness |
| 85162 |  | Cerebellar or brain stem laceration without mention of open intracranial wound, with brief [less than 1 hour] loss of consciousness |
| 85163 |  | Cerebellar or brain stem laceration without mention of open intracranial wound, with moderate [1-24 hours] loss of consciousness |
| 85164 |  | Cerebellar or brain stem laceration without mention of open intracranial wound, with prolonged [more than 24 hours] loss of consciousness and return to pre-existing conscious level |
| 85165 |  | Cerebellar or brain stem laceration without mention of open intracranial wound, with prolonged [more than 24 hours] loss of consciousness without return to pre-existing conscious level |
| 85166 |  | Cerebellar or brain stem laceration without mention of open intracranial wound, with loss of consciousness of unspecified duration |
| 85169 |  | Cerebellar or brain stem laceration without mention of open intracranial wound, with concussion, unspecified |
| 85170 |  | Cerebellar or brain stem laceration with open intracranial wound, unspecified state of consciousness |
| 85171 |  | Cerebellar or brain stem laceration with open intracranial wound, with no loss of consciousness |
| 85172 |  | Cerebellar or brain stem laceration with open intracranial wound, with brief [less than one hour] loss of consciousness |
| 85173 |  | Cerebellar or brain stem laceration with open intracranial wound, with moderate [1-24 hours] loss of consciousness |
| 85174 |  | Cerebellar or brain stem laceration with open intracranial wound, with prolonged [more than 24 hours] loss of consciousness and return to pre-existing conscious level |
| 85175 |  | Cerebellar or brain stem laceration with open intracranial wound, with prolonged [more than 24 hours] loss of consciousness without return to pre-existing conscious level |
| 85176 |  | Cerebellar or brain stem laceration with open intracranial wound, with loss of consciousness of unspecified duration |
| 85179 |  | Cerebellar or brain stem laceration with open intracranial wound, with concussion, unspecified |
| 85180 |  | Other and unspecified cerebral laceration and contusion, without mention of open intracranial wound, unspecified state of consciousness |
| 85181 |  | Other and unspecified cerebral laceration and contusion, without mention of open intracranial wound, with no loss of consciousness |
| 85182 |  | Other and unspecified cerebral laceration and contusion, without mention of open intracranial wound, with brief [less than one hour] loss of consciousness |
| 85183 |  | Other and unspecified cerebral laceration and contusion, without mention of open intracranial wound, with moderate [1-24 hours] loss of consciousness |
| 85184 |  | Other and unspecified cerebral laceration and contusion, without mention of open intracranial wound, with prolonged [more than 24 hours] loss of consciousness and return to pre- existing conscious level |
| 85185 |  | Other and unspecified cerebral laceration and contusion, without mention of open intracranial wound, with prolonged [more than 24 hours] loss of consciousness without return to pre-existing conscious level |
| 85186 |  | Other and unspecified cerebral laceration and contusion, without mention of open intracranial wound, with loss of consciousness of unspecified duration |
| 85189 |  | Other and unspecified cerebral laceration and contusion, without mention of open intracranial wound, with concussion, unspecified |
| 85190 |  | Other and unspecified cerebral laceration and contusion, with open intracranial wound, unspecified state of consciousness |
| 85191 |  | Other and unspecified cerebral laceration and contusion, with open intracranial wound, with no loss of consciousness |
| 85192 |  | Other and unspecified cerebral laceration and contusion, with open intracranial wound, with brief [less than one hour] loss of consciousness |
| 80039 |  | Closed fracture of vault of skull with other and unspecified intracranial hemorrhage, with concussion, unspecified |
| 80040 |  | Closed fracture of vault of skull with intracranial injury of other and unspecified nature, unspecified state of consciousness |
| 80041 |  | Closed fracture of vault of skull with intracranial injury of other and unspecified nature, with no loss of consciousness |
| 80042 |  | Closed fracture of vault of skull with intracranial injury of other and unspecified nature, with brief [less than one hour] loss of consciousness |
| 80043 |  | Closed fracture of vault of skull with intracranial injury of other and unspecified nature, with moderate [1-24 hours] loss of consciousness |
| 80044 |  | Closed fracture of vault of skull with intracranial injury of other and unspecified nature, with prolonged [more than 24 hours] loss of consciousness and return to pre-existing conscious level |
| 80045 |  | Closed fracture of vault of skull with intracranial injury of other and unspecified nature, with prolonged [more than 24 hours] loss of consciousness, without return to pre-existing conscious level |
| 80046 |  | Closed fracture of vault of skull with intracranial injury of other and unspecified nature, with loss of consciousness of unspecified duration |
| 80049 |  | Closed fracture of vault of skull with intracranial injury of other and unspecified nature, with concussion, unspecified |
| 80050 |  | Open fracture of vault of skull without mention of intracranial injury, unspecified state of consciousness |
| 80051 |  | Open fracture of vault of skull without mention of intracranial injury, with no loss of consciousness |
| 80052 |  | Open fracture of vault of skull without mention of intracranial injury, with brief [less than one hour] loss of consciousness |
| 80053 |  | Open fracture of vault of skull without mention of intracranial injury, with moderate [1-24 hours] loss of consciousness |
| 80054 |  | Open fracture of vault of skull without mention of intracranial injury, with prolonged [more than 24 hours] loss of consciousness and return to pre-existing conscious level |
| 80055 |  | Open fracture of vault of skull without mention of intracranial injury, with prolonged [more than 24 hours] loss of consciousness, without return to pre-existing conscious level |
| 80056 |  | Open fracture of vault of skull without mention of intracranial injury, with loss of consciousness of unspecified duration |
| 80059 |  | Open fracture of vault of skull without mention of intracranial injury, with concussion, unspecified |
| 80060 |  | Open fracture of vault of skull with cerebral laceration and contusion, unspecified state of consciousness |
| 80061 |  | Open fracture of vault of skull with cerebral laceration and contusion, with no loss of consciousness |
| 80062 |  | Open fracture of vault of skull with cerebral laceration and contusion, with brief [less than one hour] loss of consciousness |
| 80063 |  | Open fracture of vault of skull with cerebral laceration and contusion, with moderate [1-24 hours] loss of consciousness |
| 80064 |  | Open fracture of vault of skull with cerebral laceration and contusion, with prolonged [more than 24 hours] loss of consciousness and return to pre-existing conscious level |
| 80065 |  | Open fracture of vault of skull with cerebral laceration and contusion, with prolonged [more than 24 hours] loss of consciousness, without return to pre-existing conscious level |
| 80066 |  | Open fracture of vault of skull with cerebral laceration and contusion, with loss of consciousness of unspecified duration |
| 80069 |  | Open fracture of vault of skull with cerebral laceration and contusion, with concussion, unspecified |
| 80070 |  | Open fracture of vault of skull with subarachnoid, subdural, and extradural hemorrhage, unspecified state of consciousness |
| 80071 |  | Open fracture of vault of skull with subarachnoid, subdural, and extradural hemorrhage, with no loss of consciousness |
| 80072 |  | Open fracture of vault of skull with subarachnoid, subdural, and extradural hemorrhage, with brief [less than one hour] loss of consciousness |
| 80073 |  | Open fracture of vault of skull with subarachnoid, subdural, and extradural hemorrhage, with moderate [1-24 hours] loss of consciousness |
| 80074 |  | Open fracture of vault of skull with subarachnoid, subdural, and extradural hemorrhage, with moderate [1-24 hours] loss of consciousness |
| 80075 |  | Open fracture of vault of skull with subarachnoid, subdural, and extradural hemorrhage, with prolonged [more than 24 hours] loss of consciousness, without return to pre-existing conscious level |
| 80076 |  | Open fracture of vault of skull with subarachnoid, subdural, and extradural hemorrhage, with loss of consciousness of unspecified duration |
| 80079 |  | Open fracture of vault of skull with subarachnoid, subdural, and extradural hemorrhage, with concussion, unspecified |
| 80080 |  | Open fracture of vault of skull with other and unspecified intracranial hemorrhage, unspecified state of consciousness |
| 80081 |  | Open fracture of vault of skull with other and unspecified intracranial hemorrhage, with no loss of consciousness |
| 80082 |  | Open fracture of vault of skull with other and unspecified intracranial hemorrhage, with brief [less than one hour] loss of |
| 80083 |  | Open fracture of vault of skull with other and unspecified intracranial hemorrhage, with moderate [1-24 hours] loss of consciousness |
| 80084 |  | Open fracture of vault of skull with other and unspecified intracranial hemorrhage, with prolonged [more than 24 hours] loss of consciousness and return to pre-existing conscious level |
| 80085 |  | Open fracture of vault of skull with other and unspecified intracranial hemorrhage, with prolonged [more than 24 hours] loss of consciousness, without return to pre-existing conscious level |
| 80086 |  | Open fracture of vault of skull with other and unspecified intracranial hemorrhage, with loss of consciousness of unspecified duration |
| 80089 |  | Open fracture of vault of skull with other and unspecified intracranial hemorrhage, with concussion, unspecified |
| 80090 |  | Open fracture of vault of skull with intracranial injury of other and unspecified nature, unspecified state of consciousness |
| 80091 |  | Open fracture of vault of skull with intracranial injury of other and unspecified nature, with no loss of consciousness |
| 80092 |  | Open fracture of vault of skull with intracranial injury of other and unspecified nature, with brief [less than one hour] loss of consciousness |
| 80093 |  | Open fracture of vault of skull with intracranial injury of other and unspecified nature, with moderate [1-24 hours] loss of consciousness |
| 80094 |  | Open fracture of vault of skull with intracranial injury of other and unspecified nature, with prolonged [more than 24 hours] loss of consciousness and return to pre-existing conscious level |
| 80095 |  | Open fracture of vault of skull with intracranial injury of other and unspecified nature, with prolonged [more than 24 hours] loss of consciousness, without return to pre-existing conscious level |
| 80096 |  | Open fracture of vault of skull with intracranial injury of other and unspecified nature, with loss of consciousness of unspecified duration |
| 80099 |  | Open fracture of vault of skull with intracranial injury of other and unspecified nature, with concussion, unspecified |
| 80100 |  | Closed fracture of base of skull without mention of intra cranial injury, unspecified state of consciousness |
| 80101 |  | Closed fracture of base of skull without mention of intra cranial injury, with no loss of consciousness |
| 80102 |  | Closed fracture of base of skull without mention of intra cranial injury, with brief [less than one hour] loss of consciousness |
| 80103 |  | Closed fracture of base of skull without mention of intra cranial injury, with moderate [1-24 hours] loss of consciousness |
| 80104 |  | Closed fracture of base of skull without mention of intra cranial injury, with prolonged [more than 24 hours] loss of consciousness and return to pre-existing conscious level |
| 80105 |  | Closed fracture of base of skull without mention of intra cranial injury, with prolonged [more than 24 hours] loss of consciousness, without return to pre-existing conscious level |
| 80106 |  | Closed fracture of base of skull without mention of intra cranial injury, with loss of consciousness of unspecified duration |
| 80109 |  | Closed fracture of base of skull without mention of intra cranial injury, with concussion, unspecified |
| 80110 |  | Closed fracture of base of skull with cerebral laceration and contusion, unspecified state of consciousness |
| 80111 |  | Closed fracture of base of skull with cerebral laceration and contusion, with no loss of consciousness |
| 80112 |  | Closed fracture of base of skull with cerebral laceration and contusion, with brief [less than one hour] loss of consciousness |
| 80113 |  | Closed fracture of base of skull with cerebral laceration and contusion, with moderate [1-24 hours] loss of consciousness |
| 80114 |  | Closed fracture of base of skull with cerebral laceration and contusion, with prolonged [more than 24 hours] loss of consciousness and return to pre-existing conscious level |
| 80115 |  | Closed fracture of base of skull with cerebral laceration and contusion, with prolonged [more than 24 hours] loss of consciousness, without return to pre-existing conscious level |
| 80116 |  | Closed fracture of base of skull with cerebral laceration and contusion, with loss of consciousness of unspecified duration |
| 80119 |  | Closed fracture of base of skull with cerebral laceration and contusion, with concussion, unspecified |
| 80120 |  | Closed fracture of base of skull with subarachnoid, subdural, and extradural hemorrhage, unspecified state of consciousness |
| 80121 |  | Closed fracture of base of skull with subarachnoid, subdural, and extradural hemorrhage, with no loss of consciousness |
| 80122 |  | Closed fracture of base of skull with subarachnoid, subdural, and extradural hemorrhage, with brief [less than one hour] loss of consciousness |
| 80123 |  | Closed fracture of base of skull with subarachnoid, subdural, and extradural hemorrhage, with moderate [1-24 hours] loss of consciousness |
| 80124 |  | Closed fracture of base of skull with subarachnoid, subdural, and extradural hemorrhage, with prolonged [more than 24 hours] loss of consciousness and return to pre-existing conscious level |
| 80300 |  | Other closed skull fracture without mention of intracranial injury, unspecified state of consciousness |
| 80301 |  | Other closed skull fracture without mention of intracranial injury, with no loss of consciousness |
| 80302 |  | Other closed skull fracture without mention of intracranial injury, with brief [less than one hour] loss of consciousness |
| 80303 |  | Other closed skull fracture without mention of intracranial injury, with moderate [1-24 hours] loss of consciousness |
| 80304 |  | Other closed skull fracture without mention of intracranial injury, with prolonged [more than 24 hours] loss of consciousness and return to pre-existing conscious level |
| 80305 |  | Other closed skull fracture without mention of intracranial injury, with prolonged [more than 24 hours] loss of consciousness, without return to pre-existing conscious level |
| 80306 |  | Other closed skull fracture without mention of intracranial injury, with loss of consciousness of unspecified duration |
| 80309 |  | Other closed skull fracture without mention of intracranial injury, with concussion, unspecified |
| 80310 |  | Other closed skull fracture with cerebral laceration and contusion, unspecified state of consciousness |
| 80311 |  | Other closed skull fracture with cerebral laceration and contusion, with no loss of consciousness |
| 80312 |  | Other closed skull fracture with cerebral laceration and contusion, with brief [less than one hour] loss of consciousness |
| 80313 |  | Other closed skull fracture with cerebral laceration and contusion, with moderate [1-24 hours] loss of consciousness |
| 80314 |  | Other closed skull fracture with cerebral laceration and contusion, with prolonged [more than 24 hours] loss of consciousness and return to pre-existing conscious level |
| 80315 |  | Other closed skull fracture with cerebral laceration and contusion, with prolonged [more than 24 hours] loss of consciousness, without return to pre-existing conscious level |
| 80316 |  | Other closed skull fracture with cerebral laceration and contusion, with loss of consciousness of unspecified duration |
| 80319 |  | Other closed skull fracture with cerebral laceration and contusion, with concussion, unspecified |
| 80320 |  | Other closed skull fracture with subarachnoid, subdural, and extradural hemorrhage, unspecified state of consciousness |
| 80321 |  | Other closed skull fracture with subarachnoid, subdural, and extradural hemorrhage, with no loss of consciousness |
| 80322 |  | Other closed skull fracture with subarachnoid, subdural, and extradural hemorrhage, with brief [less than one hour] loss of consciousness |
| 80323 |  | Other closed skull fracture with subarachnoid, subdural, and extradural hemorrhage, with moderate [1-24 hours] loss of consciousness |
| 80324 |  | Other closed skull fracture with subarachnoid, subdural, and extradural hemorrhage, with prolonged [more than 24 hours] loss of consciousness and return to pre-existing conscious level |
| 80325 |  | Other closed skull fracture with subarachnoid, subdural, and extradural hemorrhage, with prolonged [more than 24 hours] loss of consciousness, without return to pre-existing conscious level |
| 80326 |  | Other closed skull fracture with subarachnoid, subdural, and extradural hemorrhage, with loss of consciousness of unspecified duration |
| 80329 |  | Other closed skull fracture with subarachnoid, subdural, and extradural hemorrhage, with concussion, unspecified |
| 80330 |  | Other closed skull fracture with other and unspecified intracranial hemorrhage, unspecified state of unconsciousness |
| 80331 |  | Other closed skull fracture with other and unspecified intracranial hemorrhage, with no loss of consciousness |
| 80332 |  | Other closed skull fracture with other and unspecified intracranial hemorrhage, with brief [less than one hour] loss of consciousness |
| 80333 |  | Other closed skull fracture with other and unspecified intracranial hemorrhage, with moderate [1-24 hours] loss of consciousness |
| 80334 |  | Other closed skull fracture with other and unspecified intracranial hemorrhage, with prolonged [more than 24 hours] loss of consciousness and return to pre-existing conscious level |
| 80335 |  | Other closed skull fracture with other and unspecified intracranial hemorrhage, with prolonged [more than 24 hours] loss of consciousness, without return to pre-existing conscious level |
| 80336 |  | Other closed skull fracture with other and unspecified intracranial hemorrhage, with loss of consciousness of unspecified duration |
| 80339 |  | Other closed skull fracture with other and unspecified intracranial hemorrhage, with concussion, unspecified |
| 80340 |  | Other closed skull fracture with intracranial injury of other and unspecified nature, unspecified state of consciousness |
| 80341 |  | Other closed skull fracture with intracranial injury of other and unspecified nature, with no loss of consciousness |
| 80342 |  | Other closed skull fracture with intracranial injury of other and unspecified nature, with brief [less than one hour] loss of consciousness |
| 80343 |  | Other closed skull fracture with intracranial injury of other and unspecified nature, with moderate [1-24 hours] loss of consciousness |
| 80344 |  | Other closed skull fracture with intracranial injury of other and unspecified nature, with prolonged [more than 24 hours] loss of consciousness and return to pre-existing conscious level |
| 80345 |  | Other closed skull fracture with intracranial injury of other and unspecified nature, with prolonged [more than 24 hours] loss of consciousness, without return to pre-existing conscious level |
| 8503 |  | Concussion with prolonged loss of consciousness and return to pre-existing conscious level |
| 85193 |  | Other and unspecified cerebral laceration and contusion, with open intracranial wound, with moderate [1-24 hours] loss of consciousness |
| 85194 |  | Other and unspecified cerebral laceration and contusion, with open intracranial wound, with prolonged [more than 24 hours] loss of consciousness and return to pre-existing conscious level |
| 85195 |  | Other and unspecified cerebral laceration and contusion, with open intracranial wound, with prolonged [more than 24 hours] loss of consciousness without return to pre-existing conscious level |
| 85196 |  | Other and unspecified cerebral laceration and contusion, with open intracranial wound, with loss of consciousness of unspecified duration |
| 85199 |  | Other and unspecified cerebral laceration and contusion, with open intracranial wound, with concussion, unspecified |
| 85200 |  | Subarachnoid hemorrhage following injury without mention of open intracranial wound, unspecified state of consciousness |
| 85201 |  | Subarachnoid hemorrhage following injury without mention of open intracranial wound, with no loss of consciousness |
| 85412 |  | Intracranial injury of other and unspecified nature with open intracranial wound, with brief [less than one hour] loss of consciousness |
| 85413 |  | Intracranial injury of other and unspecified nature with open intracranial wound, with moderate [1-24 hours] loss of consciousness |
| 85414 |  | Intracranial injury of other and unspecified nature with open intracranial wound, with prolonged [more than 24 hours] loss of consciousness and return to pre-existing conscious level |
| 85415 |  | Intracranial injury of other and unspecified nature with open intracranial wound, with prolonged [more than 24 hours] loss of consciousness without return to pre-existing conscious level |
| 85416 |  | Intracranial injury of other and unspecified nature with open intracranial wound, with loss of consciousness of unspecified duration |
| 85419 |  | Intracranial injury of other and unspecified nature with open intracranial wound, with concussion, unspecified |
| 80411 |  | Closed fractures involving skull or face with other bones, with cerebral laceration and contusion, with no loss of consciousness |
| 80420 |  | Closed fractures involving skull or face with other bones with subarachnoid, subdural, and extradural hemorrhage, unspecified state of consciousness |
| 80421 |  | Closed fractures involving skull or face with other bones with subarachnoid, subdural, and extradural hemorrhage, with no loss of consciousness |
| 80422 |  | Closed fractures involving skull or face with other bones with subarachnoid, subdural, and extradural hemorrhage, with brief [less than one hour] loss of consciousness |
| 80423 |  | Closed fractures involving skull or face with other bones with subarachnoid, subdural, and extradural hemorrhage, with moderate [1-24 hours] loss of consciousness |
| 80424 |  | Closed fractures involving skull or face with other bones with subarachnoid, subdural, and extradural hemorrhage, with prolonged [more than 24 hours] loss of consciousness and return to pre-existing conscious level |
| 80425 |  | Closed fractures involving skull or face with other bones with subarachnoid, subdural, and extradural hemorrhage, with prolonged [more than 24 hours] loss of consciousness, without return to pre-existing conscious level |
| 80426 |  | Closed fractures involving skull or face with other bones with subarachnoid, subdural, and extradural hemorrhage, with loss of consciousness of unspecified duration |
| 80429 |  | Closed fractures involving skull or face with other bones with subarachnoid, subdural, and extradural hemorrhage, with concussion, unspecified |
| 80430 |  | Closed fractures involving skull or face with other bones, with other and unspecified intracranial hemorrhage, unspecified state of consciousness |
| 80431 |  | Closed fractures involving skull or face with other bones, with other and unspecified intracranial hemorrhage, with no loss of consciousness |
| 80432 |  | Closed fractures involving skull or face with other bones, with other and unspecified intracranial hemorrhage, with brief [less than one hour] loss of consciousness |
| 80433 |  | Closed fractures involving skull or face with other bones, with other and unspecified intracranial hemorrhage, with moderate [1-24 hours] loss of consciousness |
| 85250 |  | Extradural hemorrhage following injury with open intracranial wound, unspecified state of consciousness |
| 85251 |  | Extradural hemorrhage following injury with open intracranial wound, with no loss of consciousness |
| 85252 |  | Extradural hemorrhage following injury with open intracranial wound, with brief [less than one hour] loss of consciousness |
| 85253 |  | Extradural hemorrhage following injury with open intracranial wound, with moderate [1-24 hours] loss of consciousness |
| 85254 |  | Extradural hemorrhage following injury with open intracranial wound, with prolonged [more than 24 hours] loss of consciousness and return to pre-existing conscious level |
| 85255 |  | Extradural hemorrhage following injury with open intracranial wound, with prolonged [more than 24 hours] loss of consciousness without return to pre-existing conscious level |
| 85256 |  | Extradural hemorrhage following injury with open intracranial wound, with loss of consciousness of unspecified duration |
| 85259 |  | Extradural hemorrhage following injury with open intracranial wound, with concussion, unspecified |
| 85300 |  | Other and unspecified intracranial hemorrhage following injury without mention of open intracranial wound, unspecified state of consciousness |
| 85301 |  | Other and unspecified intracranial hemorrhage following injury without mention of open intracranial wound, with no loss of consciousness |
| 85302 |  | Other and unspecified intracranial hemorrhage following injury without mention of open intracranial wound, with brief [less than one hour] loss of consciousness |
| 85303 |  | Other and unspecified intracranial hemorrhage following injury without mention of open intracranial wound, with moderate [1-24 hours] loss of consciousness |
| 85304 |  | Other and unspecified intracranial hemorrhage following injury without mention of open intracranial wound, with prolonged [more than 24 hours] loss of consciousness and return to pre- existing conscious level |
| 85305 |  | Other and unspecified intracranial hemorrhage following injury without mention of open intracranial wound, with prolonged [more than 24 hours] loss of consciousness without return to pre-existing conscious level |
| 85306 |  | Other and unspecified intracranial hemorrhage following injury without mention of open intracranial wound, with loss of consciousness of unspecified duration |
| 85309 |  | Other and unspecified intracranial hemorrhage following injury without mention of open intracranial wound, with concussion, unspecified |
| 85310 |  | Other and unspecified intracranial hemorrhage following injury with open intracranial wound, unspecified state of consciousness |
| 85311 |  | Other and unspecified intracranial hemorrhage following injury with open intracranial wound, with no loss of consciousness |
| 85312 |  | Other and unspecified intracranial hemorrhage following injury with open intracranial wound, with brief [less than one hour] loss of consciousness |
| 85313 |  | Other and unspecified intracranial hemorrhage following injury with open intracranial wound, with moderate [1-24 hours] loss of consciousness |
| 85314 |  | Other and unspecified intracranial hemorrhage following injury with open intracranial wound, with prolonged [more than 24 hours] loss of consciousness and return to pre-existing conscious level |
| 85315 |  | Other and unspecified intracranial hemorrhage following injury with open intracranial wound, with prolonged [more than 24 hours] loss of consciousness without return to pre-existing conscious level |
| 85316 |  | Other and unspecified intracranial hemorrhage following injury with open intracranial wound, with loss of consciousness of unspecified duration |
| 85319 |  | Other and unspecified intracranial hemorrhage following injury with open intracranial wound, with concussion, unspecified |
| 85400 |  | Intracranial injury of other and unspecified nature without mention of open intracranial wound, unspecified state of consciousness |
| 85401 |  | Intracranial injury of other and unspecified nature without mention of open intracranial wound, with no loss of consciousness |
| 85402 |  | Intracranial injury of other and unspecified nature without mention of open intracranial wound, with brief [less than one hour] loss of consciousness |
| 85403 |  | Intracranial injury of other and unspecified nature without mention of open intracranial wound, with moderate [1-24 hours] loss of consciousness |
| 85404 |  | Intracranial injury of other and unspecified nature without mention of open intracranial wound, with prolonged [more than 24 hours] loss of consciousness and return to pre-existing conscious level |
| 85405 |  | Intracranial injury of other and unspecified nature without mention of open intracranial wound, with prolonged [more than 24 hours] loss of consciousness without return to pre-existing conscious level |
| 85406 |  | Intracranial injury of other and unspecified nature without mention of open intracranial wound, with loss of consciousness of unspecified duration |
| 85409 |  | Intracranial injury of other and unspecified nature without mention of open intracranial wound, with concussion, unspecified |
| 85410 |  | Intracranial injury of other and unspecified nature with open intracranial wound, unspecified state of consciousness |
| 85411 |  | Intracranial injury of other and unspecified nature with open intracranial wound, with no loss of consciousness |
| 85412 |  | Intracranial injury of other and unspecified nature with open intracranial wound, with brief [less than one hour] loss of consciousness |
| 85413 |  | Intracranial injury of other and unspecified nature with open intracranial wound, with moderate [1-24 hours] loss of consciousness |
| 85414 |  | Intracranial injury of other and unspecified nature with open intracranial wound, with prolonged [more than 24 hours] loss of consciousness and return to pre-existing conscious level |
| 85415 |  | Intracranial injury of other and unspecified nature with open intracranial wound, with prolonged [more than 24 hours] loss of consciousness without return to pre-existing conscious level |
| 85416 |  | Intracranial injury of other and unspecified nature with open intracranial wound, with loss of consciousness of unspecified duration |
| 85419 |  | Intracranial injury of other and unspecified nature with open intracranial wound, with concussion, unspecified |
| 80434 |  | Closed fractures involving skull or face with other bones, with other and unspecified intracranial hemorrhage, with prolonged [more than 24 hours] loss of consciousness and return to pre- existing conscious level |
| 80435 |  | Closed fractures involving skull or face with other bones, with other and unspecified intracranial hemorrhage, with prolonged [more than 24 hours] loss of consciousness, without return to pre-existing conscious level |
| 80436 |  | Closed fractures involving skull or face with other bones, with other and unspecified intracranial hemorrhage, with loss of consciousness of unspecified duration |
| 80439 |  | Closed fractures involving skull or face with other bones, with other and unspecified intracranial hemorrhage, with concussion, unspecified |
| 80440 |  | Closed fractures involving skull or face with other bones, with intracranial injury of other and unspecified nature, unspecified state of consciousness |
| 80441 |  | Closed fractures involving skull or face with other bones, with intracranial injury of other and unspecified nature, with no loss of consciousness |
| 80442 |  | Closed fractures involving skull or face with other bones, with intracranial injury of other and unspecified nature, with brief [less than one hour] loss of consciousness |
| 80443 |  | Closed fractures involving skull or face with other bones, with intracranial injury of other and unspecified nature, with moderate [1-24 hours] loss of consciousness |
| 80444 |  | Closed fractures involving skull or face with other bones, with intracranial injury of other and unspecified nature, with prolonged [more than 24 hours] loss of consciousness and return to pre-existing conscious level |
| 80445 |  | Closed fractures involving skull or face with other bones, with intracranial injury of other and unspecified nature, with prolonged [more than 24 hours] loss of consciousness, without return to pre-existing conscious level |
| 80446 |  | Closed fractures involving skull or face with other bones, with intracranial injury of other and unspecified nature, with loss of consciousness of unspecified duration |
| 80449 |  | Closed fractures involving skull or face with other bones, with intracranial injury of other and unspecified nature, with concussion, unspecified |
| 80450 |  | Open fractures involving skull or face with other bones, without mention of intracranial injury, unspecified state of consciousness |
| 80451 |  | Open fractures involving skull or face with other bones, without mention of intracranial injury, with no loss of consciousness |
| 80452 |  | Open fractures involving skull or face with other bones, without mention of intracranial injury, with brief [less than one hour] loss of consciousness |
| 80453 |  | Open fractures involving skull or face with other bones, without mention of intracranial injury, with moderate [1-24 hours] loss of consciousness |
| 80454 |  | Open fractures involving skull or face with other bones, without mention of intracranial injury, with prolonged [more than 24 hours] loss of consciousness and return to pre-existing conscious level |
| 80455 |  | Open fractures involving skull or face with other bones, without mention of intracranial injury, with prolonged [more than 24 hours] loss of consciousness, without return to pre-existing conscious level |
| 80456 |  | Open fractures involving skull or face with other bones, without mention of intracranial injury, with loss of consciousness of unspecified duration |
| 80459 |  | Open fractures involving skull or face with other bones, without mention of intracranial injury, with concussion, unspecified |
| 80460 |  | Open fractures involving skull or face with other bones, with cerebral laceration and contusion, unspecified state of consciousness |
| 80461 |  | Open fractures involving skull or face with other bones, with cerebral laceration and contusion, with no loss of consciousness |
| 80462 |  | Open fractures involving skull or face with other bones, with cerebral laceration and contusion, with brief [less than one hour] loss of consciousness |
| 80463 |  | Open fractures involving skull or face with other bones, with cerebral laceration and contusion, with moderate [1-24 hours] loss of consciousness |
| 80464 |  | Open fractures involving skull or face with other bones, with cerebral laceration and contusion, with prolonged [more than 24 hours] loss of consciousness and return to pre-existing conscious level |
| 80465 |  | Open fractures involving skull or face with other bones, with cerebral laceration and contusion, with prolonged [more than 24 hours] loss of consciousness, without return to pre-existing conscious level |
| 80466 |  | Open fractures involving skull or face with other bones, with cerebral laceration and contusion, with loss of consciousness of unspecified duration |
| 80469 |  | Open fractures involving skull or face with other bones, with cerebral laceration and contusion, with concussion, unspecified |
| 80470 |  | Open fractures involving skull or face with other bones with subarachnoid, subdural, and extradural hemorrhage, unspecified state of consciousness |
| 80471 |  | Open fractures involving skull or face with other bones with subarachnoid, subdural, and extradural hemorrhage, with no loss of consciousness |
| 80472 |  | Open fractures involving skull or face with other bones with subarachnoid, subdural, and extradural hemorrhage, with brief [less than one hour] loss of consciousness |
| 80473 |  | Open fractures involving skull or face with other bones with subarachnoid, subdural, and extradural hemorrhage, with moderate [1-24 hours] loss of consciousness |
| 85103 |  | Cortex (cerebral) contusion without mention of open intracranial wound, with moderate [1-24 hours] loss of consciousness |
| 85104 |  | Cortex (cerebral) contusion without mention of open intracranial wound, with prolonged [more than 24 hours] loss of consciousness and return to pre-existing conscious level |
| 85105 |  | Cortex (cerebral) contusion without mention of open intracranial wound, with prolonged [more than 24 hours] loss of consciousness without return to pre-existing conscious level |
| 85106 |  | Cortex (cerebral) contusion without mention of open intracranial wound, with loss of consciousness of unspecified duration |
| 80346 |  | Other closed skull fracture with intracranial injury of other and unspecified nature, with loss of consciousness of unspecified duration |
| 80349 |  | Other closed skull fracture with intracranial injury of other and unspecified nature, with concussion, unspecified |
| 80350 |  | Other open skull fracture without mention of injury, unspecified state of consciousness |
| 80351 |  | Other open skull fracture without mention of intracranial injury, with no loss of consciousness |
| 80352 |  | Other open skull fracture without mention of intracranial injury, with brief [less than one hour] loss of consciousness |
| 80353 |  | Other open skull fracture without mention of intracranial injury, with moderate [1-24 hours] loss of consciousness |
| 80354 |  | Other open skull fracture without mention of intracranial injury, with prolonged [more than 24 hours] loss of consciousness and return to pre-existing conscious level |
| 80355 |  | Other open skull fracture without mention of intracranial injury, with prolonged [more than 24 hours] loss of consciousness, without return to pre-existing conscious level |
| 80356 |  | Other open skull fracture without mention of intracranial injury, with loss of consciousness of unspecified duration |
| 80359 |  | Other open skull fracture without mention of intracranial injury, with concussion, unspecified |
| 80360 |  | Other open skull fracture with cerebral laceration and contusion, unspecified state of consciousness |
| 80361 |  | Other open skull fracture with cerebral laceration and contusion, with no loss of consciousness |
| 80362 |  | Other open skull fracture with cerebral laceration and contusion, with brief [less than one hour] loss of consciousness |
| 80363 |  | Other open skull fracture with cerebral laceration and contusion, with moderate [1-24 hours] loss of consciousness |
| 80364 |  | Other open skull fracture with cerebral laceration and contusion, with prolonged [more than 24 hours] loss of consciousness and return to pre-existing conscious level |
| 80365 |  | Other open skull fracture with cerebral laceration and contusion, with prolonged [more than 24 hours] loss of consciousness, without return to pre-existing conscious level |
| 80366 |  | Other open skull fracture with cerebral laceration and contusion, with loss of consciousness of unspecified duration |
| 80369 |  | Other open skull fracture with cerebral laceration and contusion, with concussion, unspecified |
| 80370 |  | Other open skull fracture with subarachnoid, subdural, and extradural hemorrhage, unspecified state of consciousness |
| 80371 |  | Other open skull fracture with subarachnoid, subdural, and extradural hemorrhage, with no loss of consciousness |
| 80372 |  | Other open skull fracture with subarachnoid, subdural, and extradural hemorrhage, with brief [less than one hour] loss of consciousness |
| 80373 |  | Other open skull fracture with subarachnoid, subdural, and extradural hemorrhage, with moderate [1-24 hours] loss of consciousness |
| 80374 |  | Other open skull fracture with subarachnoid, subdural, and extradural hemorrhage, with prolonged [more than 24 hours] loss of consciousness and return to pre-existing conscious level |
| 80375 |  | Other open skull fracture with subarachnoid, subdural, and extradural hemorrhage, with prolonged [more than 24 hours] loss of consciousness, without return to pre-existing conscious level |
| 80376 |  | Other open skull fracture with subarachnoid, subdural, and extradural hemorrhage, with loss of consciousness of unspecified duration |
| 80379 |  | Other open skull fracture with subarachnoid, subdural, and extradural hemorrhage, with concussion, unspecified |
| 80380 |  | Other open skull fracture with other and unspecified intracranial hemorrhage, unspecified state of consciousness |
| 80381 |  | Other open skull fracture with other and unspecified intracranial hemorrhage, with no loss of consciousness |
| 80382 |  | Other open skull fracture with other and unspecified intracranial hemorrhage, with brief [less than one hour] loss of consciousness |
| 80383 |  | Other open skull fracture with other and unspecified intracranial hemorrhage, with moderate [1-24 hours] loss of consciousness |
| 80384 |  | Other open skull fracture with other and unspecified intracranial hemorrhage, with prolonged [more than 24 hours] loss of consciousness and return to pre-existing conscious level |
| 80385 |  | Other open skull fracture with other and unspecified intracranial hemorrhage, with prolonged [more than 24 hours] loss of consciousness, without return to pre-existing conscious level |
| 80386 |  | Other open skull fracture with other and unspecified intracranial hemorrhage, with loss of consciousness of unspecified duration |
| 80389 |  | Other open skull fracture with other and unspecified intracranial hemorrhage, with concussion, unspecified |
| 80390 |  | Other open skull fracture with intracranial injury of other and unspecified nature, unspecified state of consciousness |
| 80391 |  | Other open skull fracture with intracranial injury of other and unspecified nature, with no loss of consciousness |
| 80392 |  | Other open skull fracture with intracranial injury of other and unspecified nature, with brief [less than one hour] loss of consciousness |
| 80393 |  | Other open skull fracture with intracranial injury of other and unspecified nature, with moderate [1-24 hours] loss of consciousness |
| 80394 |  | Other open skull fracture with intracranial injury of other and unspecified nature, with prolonged [more than 24 hours] loss of consciousness and return to pre-existing conscious level |
| 80395 |  | Other open skull fracture with intracranial injury of other and unspecified nature, with prolonged [more than 24 hours] loss of consciousness, without return to pre-existing conscious level |
| 80396 |  | Other open skull fracture with intracranial injury of other and unspecified nature, with loss of consciousness of unspecified duration |
| 80399 |  | Other open skull fracture with intracranial injury of other and unspecified nature, with concussion, unspecified |
| 80400 |  | Closed fractures involving skull or face with other bones, without mention of intracranial injury, unspecified state of consciousness |
| 80401 |  | Closed fractures involving skull or face with other bones, without mention of intracranial injury, with no loss of consciousness |
| 80402 |  | Closed fractures involving skull or face with other bones, without mention of intracranial injury, with brief [less than one hour] loss of consciousness |
| 80403 |  | Closed fractures involving skull or face with other bones, without mention of intracranial injury, with moderate [1-24 hours] loss of consciousness |
| 80404 |  | Closed fractures involving skull or face with other bones, without mention or intracranial injury, with prolonged [more than 24 hours] loss of consciousness and return to pre-existing conscious level |
| 80405 |  | Closed fractures involving skull of face with other bones, without mention of intracranial injury, with prolonged [more than 24 hours] loss of consciousness, without return to pre-existing conscious level |
| 80406 |  | Closed fractures involving skull of face with other bones, without mention of intracranial injury, with loss of consciousness of unspecified duration |
| 80409 |  | Closed fractures involving skull of face with other bones, without mention of intracranial injury, with concussion, unspecified |
| 80410 |  | Closed fractures involving skull or face with other bones, with cerebral laceration and contusion, unspecified state of consciousness |
| 80474 |  | Open fractures involving skull or face with other bones with subarachnoid, subdural, and extradural hemorrhage, with prolonged [more than 24 hours] loss of consciousness and return to pre-existing conscious level |
| 80475 |  | Open fractures involving skull or face with other bones with subarachnoid, subdural, and extradural hemorrhage, with prolonged [more than 24 hours] loss of consciousness, without return to pre-existing conscious level |
| 80476 |  | Open fractures involving skull or face with other bones with subarachnoid, subdural, and extradural hemorrhage, with loss of consciousness of unspecified duration |
| 80479 |  | Open fractures involving skull or face with other bones with subarachnoid, subdural, and extradural hemorrhage, with concussion, unspecified |
| 80480 |  | Open fractures involving skull or face with other bones, with other and unspecified intracranial hemorrhage, unspecified state of consciousness |
| 80481 |  | Open fractures involving skull or face with other bones, with other and unspecified intracranial hemorrhage, with no loss of consciousness |
| 80482 |  | Open fractures involving skull or face with other bones, with other and unspecified intracranial hemorrhage, with brief [less than one hour] loss of consciousness |
| 80483 |  | Open fractures involving skull or face with other bones, with other and unspecified intracranial hemorrhage, with moderate [1-24 hours] loss of consciousness |
| 80484 |  | Open fractures involving skull or face with other bones, with other and unspecified intracranial hemorrhage, with prolonged [more than 24 hours] loss of consciousness and return to pre-existing conscious level |
| 80485 |  | Open fractures involving skull or face with other bones, with other and unspecified intracranial hemorrhage, with prolonged [more than 24 hours] loss consciousness, without return to pre-existing conscious level |
| 80486 |  | Open fractures involving skull or face with other bones, with other and unspecified intracranial hemorrhage, with loss of consciousness of unspecified duration |
| 80489 |  | Open fractures involving skull or face with other bones, with other and unspecified intracranial hemorrhage, with concussion, unspecified |
| 80490 |  | Open fractures involving skull or face with other bones, with intracranial injury of other and unspecified nature, unspecified state of consciousness |
| 80491 |  | Open fractures involving skull or face with other bones, with intracranial injury of other and unspecified nature, with no loss of consciousness |
| 80492 |  | Open fractures involving skull or face with other bones, with intracranial injury of other and unspecified nature, with brief [less than one hour] loss of consciousness |
| 80493 |  | Open fractures involving skull or face with other bones, with intracranial injury of other and unspecified nature, with moderate [1-24 hours] loss of consciousness |
| 80494 |  | Open fractures involving skull or face with other bones, with intracranial injury of other and unspecified nature, with prolonged [more than 24 hours] loss of consciousness and return to pre-existing conscious level |
| 80495 |  | Open fractures involving skull or face with other bones, with intracranial injury of other and unspecified nature, with prolonged [more than 24 hours] loss of consciousness without return to pre-existing conscious level |
| 80496 |  | Open fractures involving skull or face with other bones, with intracranial injury of other and unspecified nature, with loss of consciousness of unspecified duration |
| 80499 |  | Open fractures involving skull or face with other bones, with intracranial injury of other and unspecified nature, with concussion, unspecified |
| 85109 |  | Cortex (cerebral) contusion without mention of open intracranial wound, with concussion, unspecified |
| 85110 |  | Cortex (cerebral) contusion with open intracranial wound, unspecified state of consciousness |
| 85111 |  | Cortex (cerebral) contusion with open intracranial wound, with no loss of consciousness |
| 85112 |  | Cortex (cerebral) contusion with open intracranial wound, with brief [less than one hour] loss of consciousness |
| 85113 |  | Cortex (cerebral) contusion with open intracranial wound, with moderate [1-24 hours] loss of consciousness |
| 85114 |  | Cortex (cerebral) contusion with open intracranial wound, with prolonged [more than 24 hours] loss of consciousness and return to pre-existing conscious level |
| 85115 |  | Cortex (cerebral) contusion with open intracranial wound, with prolonged [more than 24 hours] loss of consciousness without return to pre-existing conscious level |
| 85116 |  | Cortex (cerebral) contusion with open intracranial wound, with loss of consciousness of unspecified duration |
| 85119 |  | Cortex (cerebral) contusion with open intracranial wound, with concussion, unspecified |
| 85120 |  | Cortex (cerebral) laceration without mention of open intracranial wound, unspecified state of consciousness |
| 85121 |  | Cortex (cerebral) laceration without mention of open intracranial wound, with no loss of consciousness |
| 85122 |  | Cortex (cerebral) laceration without mention of open intracranial wound, with brief [less than one hour] loss of consciousness |
| 85123 |  | Cortex (cerebral) laceration without mention of open intracranial wound, with moderate [1-24 hours] loss of consciousness |
| 85124 |  | Cortex (cerebral) laceration without mention of open intracranial wound, with prolonged [more than 24 hours] loss of consciousness and return to pre-existing conscious level |
| 85125 |  | Cortex (cerebral) laceration without mention of open intracranial wound, with prolonged [more than 24 hours] loss of consciousness |
| 85126 |  | Cortex (cerebral) laceration without mention of open intracranial wound, with loss of consciousness of unspecified duration |
| 85129 |  | Cortex (cerebral) laceration without mention of open intracranial wound, with concussion, unspecified |
| 85130 |  | Cortex (cerebral) laceration with open intracranial wound, unspecified state of consciousness |
| 85131 |  | Cortex (cerebral) laceration with open intracranial wound, with no loss of consciousness |
| 85132 |  | Cortex (cerebral) laceration with open intracranial wound, with brief [less than one hour] loss of consciousness |
| 85133 |  | Cortex (cerebral) laceration with open intracranial wound, with moderate [1-24 hours] loss of consciousness |
| 85134 |  | Cortex (cerebral) laceration with open intracranial wound, with prolonged [more than 24 hours] loss of consciousness and return to pre-existing conscious level |
| 85135 |  | Cortex (cerebral) laceration with open intracranial wound, with prolonged [more than 24 hours] loss of consciousness without return to pre-existing conscious level |
| 85136 |  | Cortex (cerebral) laceration with open intracranial wound, with loss of consciousness of unspecified duration |
| 85139 |  | Cortex (cerebral) laceration with open intracranial wound, with concussion, unspecified |
| 85140 |  | Cerebellar or brain stem contusion without mention of open intracranial wound, unspecified state of consciousness |
| 85141 |  | Cerebellar or brain stem contusion without mention of open intracranial wound, with no loss of consciousness |
| 85142 |  | Cerebellar or brain stem contusion without mention of open intracranial wound, with brief [less than one hour] loss of consciousness |
| 85143 |  | Cerebellar or brain stem contusion without mention of open intracranial wound, with moderate [1-24 hours] loss of consciousness |
| 85144 |  | Cerebellar or brain stem contusion without mention of open intracranial wound, with prolonged [more than 24 hours] loss consciousness and return to pre-existing conscious level |
| 85145 |  | Cerebellar or brain stem contusion without mention of open intracranial wound, with prolonged [more than 24 hours] loss of consciousness without return to pre-existing conscious level |
| 85146 |  | Cerebellar or brain stem contusion without mention of open intracranial wound, with loss of consciousness of unspecified duration |
| 85149 |  | Cerebellar or brain stem contusion without mention of open intracranial wound, with concussion, unspecified |
| 85150 |  | Cerebellar or brain stem contusion with open intracranial wound, unspecified state of consciousness |
| 85151 |  | Cerebellar or brain stem contusion with open intracranial wound, with no loss of consciousness |
| 85152 |  | Cerebellar or brain stem contusion with open intracranial wound, with brief [less than one hour] loss of consciousness |
| 85153 |  | Cerebellar or brain stem contusion with open intracranial wound, with moderate [1-24 hours] loss of consciousness |
