# Supplementary material for: Development and Validation of a Nomogram for the Prediction of Hospital Mortality of Patients With Encephalopathy Caused by Microbial Infection: A Retrospective Cohort Study
Source: Front Microbiol. 2021 Aug 19;12:737066. doi: 10.3389/fmicb.2021.737066 (PMC8417384; doi:10.3389/fmicb.2021.737066)
Supplement: Supplementary Material 1 — Exclusion of patients with traumatic injury from the MIMIC III database according to ICD-9 codes. [file Data_Sheet_1.zip › Supplementary Material 11.docx]

**Supplementary material 11^[[1]](#footnote-1)^** Organ failure was defined by a combination of ICD-9-CM and CPT codes, as outlined in the Appendix”[1]

Appendix. ICD-9-CM or CPT-Based Classification of Acute Organ Dysfunction

| Type of Organ Failure and Code — Description |
| --- |
| Respiratory  51881 — Acute respiratory failure  51882 — Acute respiratory distress syndrome  51885 — Acute respiratory distress syndrome after shock or trauma  78609 — Respiratory insufficiency  7991 — Respiratory arrest  967 — Ventilator management |
| Cardiovascular  4580 — Hypotension, postural  7855 — Shock  785.51 — Shock, cardiogenic  785.59 — Shock, circulatory or septic  4588 — Hypotension, specified type, not elsewhere classified  4589 — Hypotension, arterial, constitutional  7963 — Hypotension, transient |
| Renal  584 — Acute renal failure  580 — Acute glomerulonephritis  585 — Renal shutdown, unspecified |
| Hepatic  570 — Acute hepatic failure or necrosis  5722 — Hepatic encephalopathy  5733 — Hepatitis, septic or unspecified |
| Hematologic  2862 — Disseminated intravascular coagulation  2866 — Purpura fulminans  2869 — Coagulopathy  2873-5 — Thrombocytopenia, primary, secondary, or unspecified |

ICD-9-CM: International Classification of Diseases, Ninth Revision, Clinical Modification;

CPT: Current Procedural Terminology.

Martin GS, Mannino DM, Eaton S, Moss M, (2003) The epidemiology of sepsis in the

United States from 1979 through 2000.The New England journal of medicine 348: 1546-1554.

1. [↑](#footnote-ref-1)
