# Supplementary material for: Development and Validation of a Nomogram for the Prediction of Hospital Mortality of Patients With Encephalopathy Caused by Microbial Infection: A Retrospective Cohort Study
Source: Front Microbiol. 2021 Aug 19;12:737066. doi: 10.3389/fmicb.2021.737066 (PMC8417384; doi:10.3389/fmicb.2021.737066)
Supplement: Supplementary Material 1 — Exclusion of patients with traumatic injury from the MIMIC III database according to ICD-9 codes. [file Data_Sheet_1.zip › Supplementary Material 13.docx]

**Supplementary material 13**: Data supplement, potential predictors by LASSO logistic regression mode

|  | 1 |
| --- | --- |
| (Intercept) | -1.65274 |
| Mingcs≥9 | 0 |
| Mingcs | 0 |
| Lung.infection | 0 |
| Lntestinal.infection | 0 |
| Urinary.tract.infection | -0.03259 |
| Catheter.related | 0 |
| Skin.and.soft.tissue | 0 |
| Abdominal.cavity | 0 |
| Hypertension | 0 |
| Diabetes | 0 |
| Cardiovascular.diseases | 0 |
| Chronic.pulmonary.disease | 0 |
| Liver.disease | 0 |
| Anemias | -0.14711 |
| Acidosis | 0 |
| Alkalosis | 0 |
| Hypovolemia | 0 |
| qsofa | 0 |
| SOFA | 0 |
| SAPSII | 0.030587 |
| Mechanical Ventilation | 0 |
| Crrtdurations | 0.321064 |
| Heartrate_max | 0 |
| Sysbp_min | -0.00913 |
| Diasbp_min | 0 |
| Meanbp_min | 0 |
| Resprate_max | 0 |
| Tempc_max | -0.00405 |
| SpO2_min | -0.00234 |
| Glucose_min | 0 |
| Glucose_max | 0 |
| Escherichia.coli | 0 |
| Klebsiella.oxytoca | 0 |
| Enterobacter | 0 |
| Proteus.mirabilis | 0 |
| Staph.aureus.coag...1 | 0 |
| Pseudomonas.aeruginosa | 0 |
| Enterococcus.sp. | 0 |
| Streptococcus | 0 |
| Candida.albicans | 0 |
| Aspergillus.fumigatus | 0 |
| Gram.negative.rod..1 | 0 |
| Yeast | 0 |
| Cryptococcus.species | 0 |
| Proteus.species | 0 |
| Stenotrophomonas..xanthomonas..maltophilia | 0 |
| Lactobacillus.species | 0 |
| Bacteroides.fragilis.group | 0 |
| Clostridium.difficile | 0 |
| Herpes.simplex.virus | 0 |
| Staphylococcus..coagulase.negative | 0 |
| Virus | 0 |
| Acinetobacter.baumannii | 0 |
| Andida.albicans..presumptive.identification | 0 |
| Positive.for.methicillin.resistant.staph.aureus | 0 |
| Pco2 | 0 |
| ph | 0 |
| WBC | 0 |
| Monocytes | 0 |
| Neutrophils | 0 |
| Lymphocytes | 0 |
| Eosinophils | 0 |
| Blood urea nitrogen | 0 |
| Creatinine | 0 |
| Aspartate aminotransferase | 0 |
| Alanine aminotransferase | 0 |
| Albumin | -0.05314 |
| Partial time | 0 |
| Irnational normalized ratio | 0.012741 |
| Partial hromboplastin time | 0.008087 |
| Potassium | 0 |
| Sodium | 0 |
| Lactate | 0.035666 |
| organ_failure | 0 |
| Respiratory failure | 0.420171 |
| Cardiovascular failure | 0 |
| Renalfailure | 0 |
| Hepatic failure | 0 |
| Hematologic failure | 0 |
| Metabolic failure | 0 |
| Neurologic failure | 0 |
| Number of organ failure: 1 | 0 |
| Number of organ failure: 2 | 0 |
| Number of organ failure: 3 | 0 |
| Number of organ failure: >3 | 0 |
| Age | 0 |
| Hemoglobin | 0 |
| Platelet | 0 |
