# Supplementary material for: Development and Validation of a Nomogram for the Prediction of Hospital Mortality of Patients With Encephalopathy Caused by Microbial Infection: A Retrospective Cohort Study
Source: Front Microbiol. 2021 Aug 19;12:737066. doi: 10.3389/fmicb.2021.737066 (PMC8417384; doi:10.3389/fmicb.2021.737066)
Supplement: Supplementary Material 1 — Exclusion of patients with traumatic injury from the MIMIC III database according to ICD-9 codes. [file Data_Sheet_1.zip › Supplementary Material 2.docx]

| **Supplementary materials 2** Exclude patients with intracerebral hemorrhage, cerebral embolism and ischemic stroke disease from the MIMIC III database according to ICD9-codes | | | | | | | | | | | | | | | | | | | | |
| --- | --- | --- | --- | --- | --- | --- | --- | --- | --- | --- | --- | --- | --- | --- | --- | --- | --- | --- | --- | --- |
| ICD9-code | |  | Description |  |  |  | |  | |  | |  | |  | |  | |  | | |
| 430 |  |  | Subarachnoid hemorrhage | | |  |  | |  | |  | |  | |  | |  | |  |  |
| 431 |  |  | Intracerebral hemorrhage | | |  |  | |  | |  | |  | |  | |  | |  |  |
| 5430 |  |  | Subarachnoid hemorrhage | | | | | | | | | | | | |  | |  | | |
| 4329 |  |  | Unspecified intracranial hemorrhage | | | | | | | | | | | | |  | |  | | |
| 4321 |  |  | Subdural hemorrhage | | | | | | | | | | |  | |  | |  | | |
| 4320 |  |  | Nontraumatic extradural hemorrhage | | | | | | | | |  | |  | |  | |  | | |
| 7670 |  |  | Subdural and cerebral hemorrhage | | | | | | | | |  | |  | |  | |  | | |
| 43411 |  |  | Cerebral embolism with cerebral infarction | | | | | | | | | | | | | | |  | | |
| 4376 |  |  | Nonpyogenic thrombosis of intracranial venous sinus | | | | | | | | | | | | | | |  | | |
| V1254 |  |  | Personal history of transient ischemic attack (TIA), and cerebral infarction without residual deficits | | | | | | | | | | | | | | |  | | |
