# Supplementary material for: Development and Validation of a Nomogram for the Prediction of Hospital Mortality of Patients With Encephalopathy Caused by Microbial Infection: A Retrospective Cohort Study
Source: Front Microbiol. 2021 Aug 19;12:737066. doi: 10.3389/fmicb.2021.737066 (PMC8417384; doi:10.3389/fmicb.2021.737066)
Supplement: Supplementary Material 1 — Exclusion of patients with traumatic injury from the MIMIC III database according to ICD-9 codes. [file Data_Sheet_1.zip › Supplementary Material 3.docx]

| **Supplementary material 3** Exclude patients with meningitis and encephalitis disease from the MIMIC III database according to ICD9-codes | | | | | | | | | | | |  |
| --- | --- | --- | --- | --- | --- | --- | --- | --- | --- | --- | --- | --- |
| ICD9-code | | Description |  |  |  |  |  |  |  |  |  | |
| 1300 |  | Tuberculous meningitis, unspecified | | | | | | | |  |  | |
| 1301 |  | Tuberculous meningitis, bacteriological or histological examination not done | | | | | | | |  |  | |
| 468 |  | Other specified slow virus infection of central nervous system | | | | | | | |  |  | |
| 469 |  | Unspecified slow virus infection of central nervous system | | | | | | | |  |  | |
| 470 |  | Meningitis due to coxsackie virus | | | | | | | |  |  | |
| 471 |  | Meningitis due to echo virus | | | | | | | |  |  | |
| 478 |  | Other specified viral meningitis | | | | | | | |  |  | |
| 479 |  | Unspecified viral meningitis | | | | | | | |  |  | |
| 491 |  | Meningitis due to adenovirus | | | | | | | |  |  | |
| 498 |  | Other specified non-arthropod-borne viral diseases of central nervous system | | | | | | | |  |  | |
| 499 |  | Unspecified non-arthropod-borne viral diseases of central nervous system | | | | | | | |  |  | |
| 520 |  | Postvaricella encephalitis | | | | | | | |  |  | |
| 530 |  | Herpes zoster with meningitis | | | | | | | |  |  | |
| 5319 |  | Herpes zoster with other nervous system complications | | | | | | | |  |  | |
| 5379 |  | Herpes zoster with other specified complications | | | | | | | |  |  | |
| 5472 |  | Herpes simplex meningitis | | | | | | | |  |  | |
| 550 |  | Postmeasles encephalitis | | | | | | | |  |  | |
| 360 |  | Meningococcal meningitis | | | | | | | |  |  | |
| 361 |  | Meningococcal encephalitis | | | | | | | |  |  | |
| 362 |  | Meningococcemia | | | | | | | |  |  | |
| 363 |  | Waterhouse-Friderichsen syndrome, meningococcal | | | | | | | |  |  | |
| 1302 |  | Tuberculous meningitis, bacteriological or histological examination unknown (at present) | | | | | | | |  |  | |
| 1303 |  | Tuberculous meningitis, tubercle bacilli found (in sputum) by microscopy | | | | | | | |  |  | |
| 1304 |  | Tuberculous meningitis, tubercle bacilli not found (in sputum) by microscopy, but found by bacterial culture | | | | | | | |  |  | |
| 1305 |  | Tuberculous meningitis, tubercle bacilli not found by bacteriological examination, but tuberculosis confirmed histologically | | | | | | | |  |  | |
| 1306 |  | Tuberculous meningitis, tubercle bacilli not found by bacteriological or histological examination, but tuberculosis confirmed by other methods [inoculation of animals] | | | | | | | |  |  | |
| 1310 |  | Tuberculoma of meninges, unspecified | | | | | | | |  |  | |
| 1311 |  | Tuberculoma of meninges, bacteriological or histological examination not done | | | | | | | |  |  | |
| 1312 |  | Tuberculoma of meninges, bacteriological or histological examination unknown (at present) | | | | | | | |  |  | |
| 1313 |  | Tuberculoma of meninges, tubercle bacilli found (in sputum) by microscopy | | | | | | | |  |  | |
| 1314 |  | Tuberculoma of meninges, tubercle bacilli not found (in sputum) by microscopy, but found by bacterial culture | | | | | | | |  |  | |
| 1315 |  | Tuberculoma of meninges, tubercle bacilli not found by bacteriological examination, but tuberculosis confirmed histologically | | | | | | | |  |  | |
| 1316 |  | Tuberculoma of meninges, tubercle bacilli not found by bacteriological or histological examination, but tuberculosis confirmed by other methods [inoculation of animals] | | | | | | | |  |  | |
| 1320 |  | Tuberculoma of brain, unspecified | | | | | | | |  |  | |
| 1321 |  | Tuberculoma of brain, bacteriological or histological examination not done | | | | | | | |  |  | |
| 1322 |  | Tuberculoma of brain, bacteriological or histological examination unknown (at present) | | | | | | | |  |  | |
| 1323 |  | Tuberculoma of brain, tubercle bacilli found (in sputum) by microscopy | | | | | | | |  |  | |
| 1324 |  | Tuberculoma of brain, tubercle bacilli not found (in sputum) by microscopy, but found by bacterial culture | | | | | | | |  |  | |
| 1325 |  | Tuberculoma of brain, tubercle bacilli not found by bacteriological examination, but tuberculosis confirmed histologically | | | | | | | |  |  | |
| 1326 |  | Tuberculoma of brain, tubercle bacilli not found by bacteriological or histological examination, but tuberculosis confirmed by other | | | | | | | |  |  | |
| 1330 |  | Tuberculous abscess of brain, unspecified | | | | | | | |  |  | |
| 1331 |  | Tuberculous abscess of brain, bacteriological or histological examination not done | | | | | | | |  |  | |
| 1332 |  | Tuberculous abscess of brain, bacteriological or histological examination unknown (at present) | | | | | | | |  |  | |
| 1333 |  | Tuberculous abscess of brain, tubercle bacilli found (in sputum) by microscopy | | | | | | | |  |  | |
| 1334 |  | Tuberculous abscess of brain, tubercle bacilli not found (in sputum) by microscopy, but found by bacterial culture | | | | | | | |  |  | |
| 1335 |  | Tuberculous abscess of brain, tubercle bacilli not found by bacteriological examination, but tuberculosis confirmed histologically | | | | | | | |  |  | |
| 1336 |  | Tuberculous abscess of brain, tubercle bacilli not found by bacteriological or histological examination, but tuberculosis confirmed by other methods [inoculation of animals] | | | | | | | |  |  | |
| 1360 |  | Tuberculous encephalitis or myelitis, unspecified | | | | | | | |  |  | |
| 1361 |  | Tuberculous encephalitis or myelitis, bacteriological or histological examination not done | | | | | | | |  |  | |
| 1362 |  | Tuberculous encephalitis or myelitis, bacteriological or histological examination unknown (at present) | | | | | | | |  |  | |
| 1363 |  | Tuberculous encephalitis or myelitis, tubercle bacilli found (in sputum) by microscopy | | | | | | | |  |  | |
| 1364 |  | Tuberculous encephalitis or myelitis, tubercle bacilli not found (in sputum) by microscopy, but found by bacterial culture | | | | | | | |  |  | |
| 1365 |  | Tuberculous encephalitis or myelitis, tubercle bacilli not found by bacteriological examination, but tuberculosis confirmed histologically | | | | | | | |  |  | |
| 1366 |  | Tuberculous encephalitis or myelitis, tubercle bacilli not found by bacteriological or histological examination, but tuberculosis confirmed by other methods [inoculation of animals] | | | | | | | |  |  | |
| 1380 |  | Other specified tuberculosis of central nervous system, unspecified | | | | | | | |  |  | |
| 1381 |  | Other specified tuberculosis of central nervous system, bacteriological or histological examination not done | | | | | | | |  |  | |
| 1382 |  | Other specified tuberculosis of central nervous system, bacteriological or histological examination unknown (at present) | | | | | | | |  |  | |
| 1383 |  | Other specified tuberculosis of central nervous system, tubercle bacilli found (in sputum) by microscopy | | | | | | | |  |  | |
| 1384 |  | Other specified tuberculosis of central nervous system, tubercle bacilli not found (in sputum) by microscopy, but found by bacterial culture | | | | | | | |  |  | |
| 1385 |  | Other specified tuberculosis of central nervous system, tubercle bacilli not found by bacteriological examination, but tuberculosis confirmed histologically | | | | | | | |  |  | |
| 1386 |  | Other specified tuberculosis of central nervous system, tubercle bacilli not found by bacteriological or histological examination, but tuberculosis confirmed by other methods [inoculation of animals] | | | | | | | |  |  | |
| 1390 |  | Unspecified tuberculosis of central nervous system, unspecified | | | | | | | |  |  | |
| 1391 |  | Unspecified tuberculosis of central nervous system, bacteriological or histological examination not done | | | | | | | |  |  | |
| 1392 |  | Unspecified tuberculosis of central nervous system, bacteriological or histological examination unknown (at present) | | | | | | | |  |  | |
| 1393 |  | Unspecified tuberculosis of central nervous system, tubercle bacilli found (in sputum) by microscopy | | | | | | | |  |  | |
| 1394 |  | Unspecified tuberculosis of central nervous system, tubercle bacilli not found (in sputum) by microscopy, but found by bacterial culture | | | | | | | |  |  | |
| 1395 |  | Unspecified tuberculosis of central nervous system, tubercle bacilli not found by bacteriological examination, but tuberculosis confirmed histologically | | | | | | | |  |  | |
| 1396 |  | Unspecified tuberculosis of central nervous system, tubercle bacilli not found by bacteriological or histological examination, but tuberculosis confirmed by other methods [inoculation of animals] | | | | | | | |  |  | |
| 1142 |  | Coccidioidal meningitis | | | | | | | |  |  | |
| 620 |  | Japanese encephalitis | | | | | | | |  |  | |
| 621 |  | Western equine encephalitis | | | | | | | |  |  | |
| 622 |  | Eastern equine encephalitis | | | | | | | |  |  | |
| 623 |  | St. Louis encephalitis | | | | | | | |  |  | |
| 624 |  | Australian encephalitis | | | | | | | |  |  | |
| 625 |  | California virus encephalitis | | | | | | | |  |  | |
| 632 |  | Central european encephalitis | | | | | | | |  |  | |
| 638 |  | Other specified tick-borne viral encephalitis | | | | | | | |  |  | |
| 3239 |  | Unspecified causes of encephalitis, myelitis, and encephalomyelitis | | | | | | | |  |  | |
| 3229 |  | Meningitis, unspecified | | | | | | | |  |  | |
| 3222 |  | Chronic meningitis | | | | | | | |  |  | |
| 3212 |  | Meningitis due to viruses not elsewhere classified | | | | | | | |  |  | |
| 3203 |  | Staphylococcal meningitis | | | | | | | |  |  | |
| 3201 |  | Pneumococcal meningitis | | | | | | | |  |  | |
