# Supplementary material for: Development and Validation of a Nomogram for the Prediction of Hospital Mortality of Patients With Encephalopathy Caused by Microbial Infection: A Retrospective Cohort Study
Source: Front Microbiol. 2021 Aug 19;12:737066. doi: 10.3389/fmicb.2021.737066 (PMC8417384; doi:10.3389/fmicb.2021.737066)
Supplement: Supplementary Material 1 — Exclusion of patients with traumatic injury from the MIMIC III database according to ICD-9 codes. [file Data_Sheet_1.zip › Supplementary Material 4.docx]

| **Supplementary material 4** Exclude patients with Epilepsy disease from the MIMIC III database according to ICD9-codes | | |
| --- | --- | --- |
| ICD9-code |  | Description |
| 34591 |  | Epilepsy, unspecified, with intractable epilepsy |
| 34570 |  | Epilepsia partialis continua, without mention of intractable epilepsy |
| 34571 |  | Epilepsia partialis continua, with intractable epilepsy |
| 34580 |  | Other forms of epilepsy and recurrent seizures, without mention of intractable epilepsy |
| 34581 |  | Other forms of epilepsy and recurrent seizures, with intractable epilepsy |
| 34590 |  | Epilepsy, unspecified, without mention of intractable epilepsy |
| 34591 |  | Epilepsy, unspecified, with intractable epilepsy |
| 64940 |  | Epilepsy complicating pregnancy, childbirth, or the puerperium, unspecified as to episode of care or not applicable |
| 64941 |  | Epilepsy complicating pregnancy, childbirth, or the puerperium, delivered, with or without mention of antepartum condition |
| 64942 |  | Epilepsy complicating pregnancy, childbirth, or the puerperium, delivered, with mention of postpartum complication |
| 64943 |  | Epilepsy complicating pregnancy, childbirth, or the puerperium, antepartum condition or complication |
| 64944 |  | Epilepsy complicating pregnancy, childbirth, or the puerperium, postpartum condition or complication |
