# Supplementary material for: Development and Validation of a Nomogram for the Prediction of Hospital Mortality of Patients With Encephalopathy Caused by Microbial Infection: A Retrospective Cohort Study
Source: Front Microbiol. 2021 Aug 19;12:737066. doi: 10.3389/fmicb.2021.737066 (PMC8417384; doi:10.3389/fmicb.2021.737066)
Supplement: Supplementary Material 1 — Exclusion of patients with traumatic injury from the MIMIC III database according to ICD-9 codes. [file Data_Sheet_1.zip › Supplementary Material 5.docx]

| **Supplementary material 5** Exclude patients with other cerebrovascular disease from the MIMIC III database according to ICD9-codes | | |
| --- | --- | --- |
| ICD9-code |  | Description |
| 3312 |  | Senile degeneration of brain |
| 3313 |  | Communicating hydrocephalus |
| 3314 |  | Obstructive hydrocephalus |
| 33189 |  | Other cerebral degeneration |
| 3319 |  | Cerebral degeneration, unspecified |
| 4378 |  | Other ill-defined cerebrovascular disease |
| 4379 |  | Unspecified cerebrovascular disease |
| 4380 |  | Unspecified cerebrovascular disease |
| 43810 |  | Late effects of cerebrovascular disease, speech and language deficit, unspecified |
