# Supplementary material for: Development and Validation of a Nomogram for the Prediction of Hospital Mortality of Patients With Encephalopathy Caused by Microbial Infection: A Retrospective Cohort Study
Source: Front Microbiol. 2021 Aug 19;12:737066. doi: 10.3389/fmicb.2021.737066 (PMC8417384; doi:10.3389/fmicb.2021.737066)
Supplement: Supplementary Material 1 — Exclusion of patients with traumatic injury from the MIMIC III database according to ICD-9 codes. [file Data_Sheet_1.zip › Supplementary Material 8.docx]

| **Supplementary material 8** Exclude patients with metabolic encephalopathy, hepatic encephalopathy,hypertensive encephalopathy,diabetes with coma, disorders of urea cycle, hypernatremia from the MIMIC III database according to ICD9-codes | | |
| --- | --- | --- |
| ICD9-codes |  | Description |
| 34831 |  | Metabolic encephalopathy |
| 5722 |  | Hepatic encephalopathy |
| 700 |  | Viral hepatitis A with hepatic coma |
| 7020 |  | Viral hepatitis B with hepatic coma, acute or unspecified, without mention of hepatitis delta |
| 7021 |  | Viral hepatitis B with hepatic coma, acute or unspecified, with hepatitis delta |
| 7022 |  | Chronic viral hepatitis B with hepatic coma without hepatitis delta |
| 7023 |  | Chronic viral hepatitis B with hepatic coma with hepatitis delta |
| 7041 |  | Acute hepatitis C with hepatic coma |
| 7042 |  | Hepatitis delta without mention of active hepatitis B disease with hepatic coma |
| 7043 |  | Hepatitis E with hepatic coma |
| 7044 |  | Chronic hepatitis C with hepatic coma |
| 7049 |  | Other specified viral hepatitis with hepatic coma |
| 7052 |  | Hepatitis delta without mention of active hepatitis B disease or hepatic coma |
| 706 |  | Unspecified viral hepatitis with hepatic coma |
| 7071 |  | Unspecified viral hepatitis C with hepatic coma |
| 2706 |  | Disorders of urea cycle metabolism |
| 2510 |  | Hypoglycemic coma |
| 4372 |  | Hypertensive encephalopathy |
